# Enhanced Efficiency of Pd(0)-Based Single Chain Polymeric Nanoparticles for *in Vitro* Prodrug Activation by Modulating the Polymer’s Microstructure

**DOI:** 10.1021/acs.nanolett.3c04466

**Published:** 2024-02-12

**Authors:** Linlin Deng, Anjana Sathyan, Catherine Adam, Asier Unciti-Broceta, Víctor Sebastian, Anja R. A. Palmans

**Affiliations:** † Laboratory for Macromolecular and Organic Chemistry, Department of Chemical Engineering and Chemistry, Eindhoven University of Technology, P.O. Box 513, 5600 MB Eindhoven, The Netherlands; ‡ Institute for Complex Molecular Systems, Eindhoven University of Technology, P.O. Box 513, 5600 MB Eindhoven, The Netherlands; § Edinburgh Cancer Research, Cancer Research UK Scotland Centre, Institute of Genetics and Cancer, University of Edinburgh, Crewe Road South, Edinburgh EH4 2XR, United Kingdom; ∥ Instituto de Nanociencia y Materiales de Aragón (INMA), CSIC-Universidad de Zaragoza, Zaragoza 50009, Spain; ⊥ Department of Chemical and Environmental Engineering, Universidad de Zaragoza, Campus Rio Ebro, 50018 Zaragoza, Spain; # Laboratorio de Microscopías Avanzadas, Universidad de Zaragoza, 50018 Zaragoza, Spain; ¶ Networking Research Center on Bioengineering, Biomaterials and Nanomedicine (CIBER-BBN), 28029 Madrid, Spain

**Keywords:** single chain polymeric nanoparticle, compartmentalization, nanocatalyst, bioorthogonal catalysis, prodrug activation, *in vitro* catalysis

## Abstract

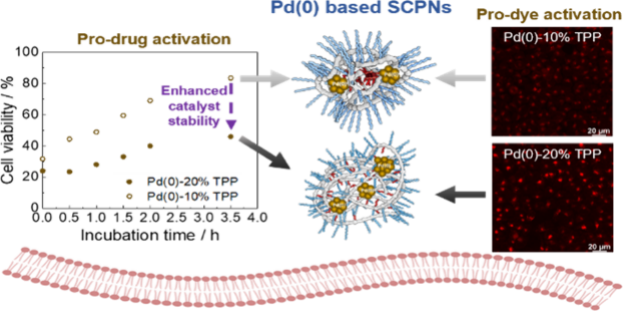

Bioorthogonal catalysis employing transition metal catalysts is a promising strategy for the *in situ* synthesis of imaging and therapeutic agents in biological environments. The transition metal Pd has been widely used as a bioorthogonal catalyst, but bare Pd poses challenges in water solubility and catalyst stability in cellular environments. In this work, Pd(0) loaded amphiphilic polymeric nanoparticles are applied to shield Pd in the presence of living cells for the *in situ* generation of a fluorescent dye and anticancer drugs. Pd(0) loaded polymeric nanoparticles prepared by the reduction of the corresponding Pd(II)-polymeric nanoparticles are highly active in the deprotection of pro-rhodamine dye and anticancer prodrugs, giving significant fluorescence enhancement and toxigenic effects, respectively, in HepG2 cells. In addition, we show that the microstructure of the polymeric nanoparticles for scaffolding Pd plays a critical role in tuning the catalytic efficiency, with the use of the ligand triphenylphosphine as a key factor for improving the catalyst stability in biological environments.

Bioorthogonal strategies to activate anticancer prodrugs have recently proved promising for developing side-effect-free cancer therapies.^1,2^ This allows the release of chemotherapeutics selectively in the tumor site either by decaging a masked pro-drug or by coupling two inactive precursors.^3,4^ There have been significant advances in the field in the past decade with the emergence of transition metal catalysts (TMCs) such as Pd(II)/(0), Ru, Au, or Cu as bioorthogonal catalysts which promoted *in situ* generation of therapeutics.^5−9^ Still, only a few of the reported bioorthogonal catalysts have succeeded *in vivo*, suggesting a significant gap between *in vitro* and *in vivo* experiments.^4,7,10^ Among the many transition metal catalysts reported to promote bioorthogonal reactions, Pd(0) displays advantageous features such as limited toxicity and versatility to perform coupling and bond cleavage reactions in complex biological media.^11−13^ However, several challenges are posed when employing naked Pd, including low water solubility and inadequate catalytic stability in the biological environment.^14,15^ To tackle these challenges, one strategy is to engineer the ligands that can complex with Pd. However, the protection and controlled delivery of the metal remain unsolved.

Inspired by enzymes where the active metal centers are often shielded by scaffolding within the protein, the Pd metal complex can be incorporated into a synthetic polymer scaffold to generate homogeneous systems that help to enhance catalyst solubility, stability, and biocompatibility while allowing targeted delivery. Thereby, these scaffolds hold great promise to develop side-effect-free cancer therapies with the potential to activate pro-drugs of chemotherapeutics specifically at the tumor site. In this respect, single chain polymeric nanoparticles (SCPNs) have been studied in great detail by us and others.^16−21^ SCPNs form from an individual polymer chain comprised of randomly distributed hydrophilic and hydrophobic groups that collapses in aqueous media into a compact, compartmentalized structure.^16,22^ Apart from shielding the metal complex from an aqueous solution, SCPNs create a hydrophobic microenvironment for localizing both substrates and catalysts, resulting in high local concentrations and thus fast kinetics of the reactions.^23−25^

Previous work within our group has elucidated the importance of optimizing the nature of the metal complexing ligand, the polymer microstructure, and the substrate hydrophobicity and indicated that triphenylphosphine (TPP) functionalized Pd(II)-based polymeric nanoparticles showed moderate activity in aqueous media toward depropargylation reactions of selected caged dyes and drugs.^19^ These investigations, however, remained limited to solution studies, hindering a comprehensive understanding of their potential translation to *in vitro* studies in the presence of cell cultures. In assessing the efficiency of Pd(II)-based polymeric nanoparticles for prodrug/dye activation, we observed a notable reduction in reaction rates when conducting catalytic reactions in cell-culture media compared to that in water.^16,19^ Consequently, to further evaluate their *in vitro* performance, enhancing the activity and stability of the catalytic system became imperative. To address these challenges, we envisaged that reduction of Pd(II) to Pd(0) within polymeric nanoparticles featuring different pendant groups including TPP ligands could increase catalytic performance and stabilize the metal within the hydrophobic compartment. As shown herein, this modification yields a more efficient catalytic system, facilitating improved assessment of their performance *in vitro*.

Four amphiphilic polymers varying in functional groups were synthesized according to previously reported protocols.^19,26^ All polymers possess a degree of polymerization of ∼200 and comprise Jeffamine M-1000 grafts to endow water solubility and biocompatibility (Scheme 1, Table S1). For the polymer containing benzene-1,3,5-tricarboxamide (BTA) grafts, the polymer is referred to as **PBTA**. As the most hydrophobic polymer, **PBTA** comprises 5% BTA, 10% TPP ligands for Pd binding, 20% dodecyl for inducing a hydrophobic collapse, and 65% Jeffamine M-1000 for endowing nanoparticles with water solubility. This microstructure is reported to form a compact and structured hydrophobic interior via 3-fold hydrogen-bonding interactions between BTA grafts.^26^ For the polymers containing the highest percentage of Jeffamine M-1000 and carboxylic acid, the polymers are named **PJ** and **PCOOH**, respectively. Both **PJ** and **PCOOH** contain 20% TPP ligands, a higher amount of triphenylphosphine ligands compared to **PBTA**. The charged polymer, **PCOOH**, was selected to compare with neutral polymers in *in vitro* studies. **PCOOH** also contains 1% Nile Red which can assist in visualizing the internalization of nanoparticles by cells and study intracellular catalysis. To study the effect of the TPP ligand, one polymer is designed without the TPP ligand and named **Pcontrol**. **Pcontrol** comprises 20% hydrophobic dodecyl groups to ensure hydrophobic collapse.

**Scheme 1:**
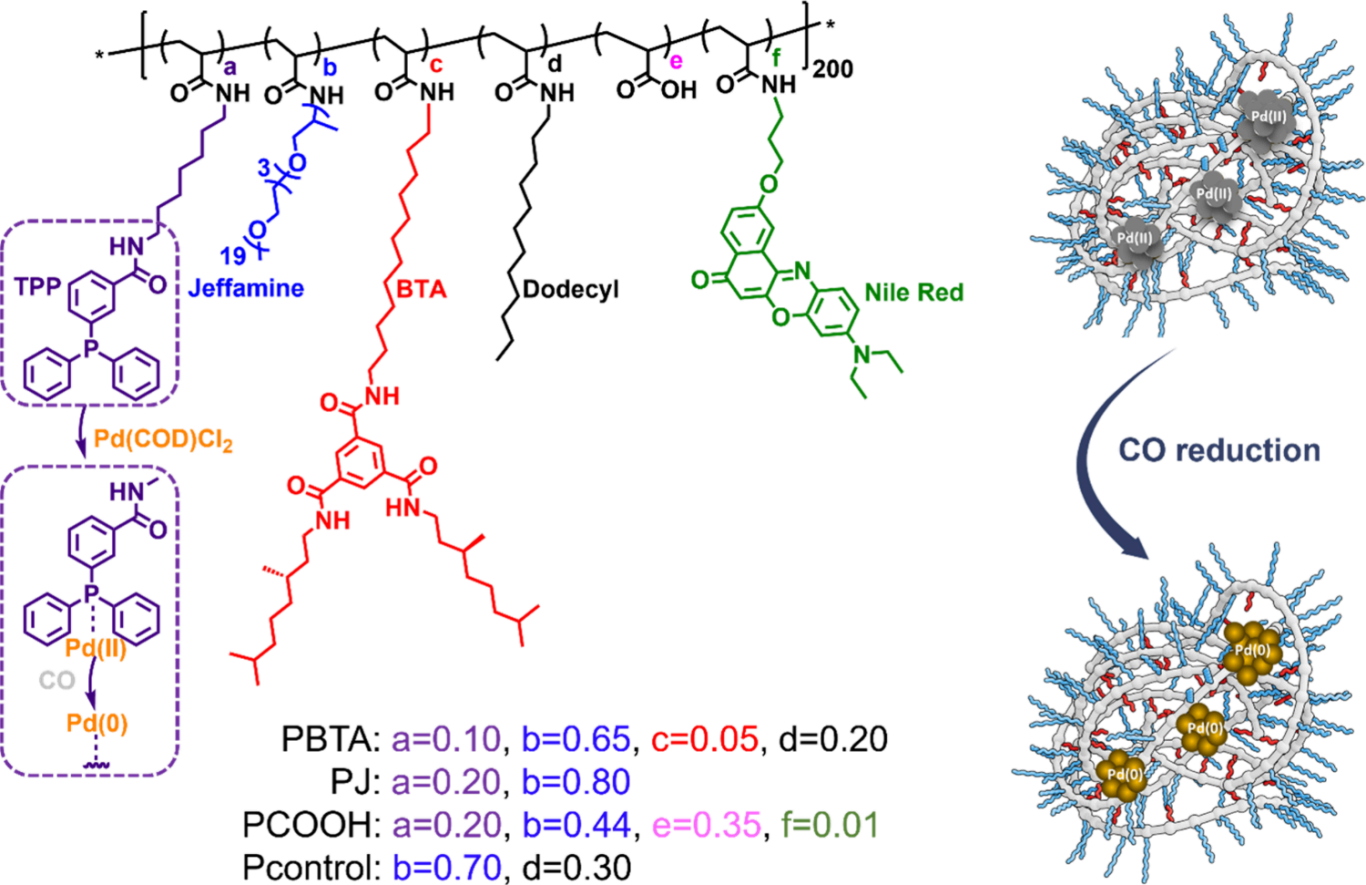
Structures of Amphiphilic Polymers **PBTA**, **PJ**, **PCOOH**, and **Pcontrol** with a Specified Ratio of Pendant Groups and Loaded with Pd(II), which Can Be Reduced into Pd(0)

TPP functionalized polymers **PBTA**, **PJ**, and **PCOOH** complex Pd(II) in Milli-Q water when using Pd(COD)Cl_2_ as the palladium source by ligand exchange ([P] = 1 mg/mL, [Pd(II)] = 210 μM). After complexing Pd(II) to the respective polymers, the system is referred to as **P@Pd(II)**. Although **Pcontrol** does not contain TPP ligands, Pd(COD)Cl_2_ is physically mixed with **Pcontrol** to prepare **Pcontrol@Pd(II)**. The obtained solutions are homogeneous, and dynamic light scattering showed the presence of small polymer nanoparticles with hydrodynamic radii *R*_H_ of 6–7 nm (see Table S1). By treating the **P@Pd(II)** polymers with CO, a mild reductant and capping agent, at 30 °C and a gas pressure of 6 bar for 60 min, Pd(II) is reduced to Pd(0). Significant color change is observed after the successful reduction (Figure S4). This technique has been successfully employed for the controlled creation of ultrathin Pd nanosheets in exosomes and agarose.^27,28^ The same protocol is here applied for preparing Pd(0)-based nanoparticles (NPs). Hereby, Pd(II) was reduced to Pd(0), affording **P@Pd(0)**, which remained homogeneous in water and showed *R*_H_ between 5 and 9 nm, albeit with a small fraction of particles with larger sizes (Table S1 and Figure S5). As a control in the absence of polymers, Pd(COD)Cl_2_ (210 μM) in Milli-Q water was also reduced to Pd(0) using the same protocol.

All **P@Pd(0)** and bare Pd(0) were analyzed using high-angle annular dark-field scanning transmission electron microscopy (HAADF-STEM) measurements. HAADF-STEM images of **PBTA@Pd(0)** show bright spherical structures (indicated by white arrows in Figure 1A) with a size of ∼5 nm in diameter. Intensity is directly proportional to the atomic number of elements which facilitates the visualization of Pd(0) NPs in contrast to the lighter atoms such as carbon which are difficult to observe. This is indicative of the formation of Pd(0) NPs with a size of 5 nm or less. **PJ@Pd(0)** also showed the presence of Pd(0) NPs with ∼5 nm size, but in this case, there was a higher fraction of particles with variable sizes (Figure 1A). **PCOOH@Pd(0)** showed highly dispersed and less defined Pd(0) NPs as observed from aggregated bright spots (Figure S6). This could be due to negatively charged carboxylate groups that strongly bind to positively charged Pd(II) precursor across the polymer backbone, which result in large aggregated, nonspherical Pd(0) NPs upon CO reduction. **Pcontrol@Pd(0)** also showed spherical Pd(0) NPs with sizes of ∼5–10 nm (Figure S6). In the absence of amphiphilic polymers, bare Pd(0) showed a trigonal sheet-like structure (Figure S8). Without amphiphilic polymers as the scaffold, bare Pd(0) precipitated after 2 weeks, while **P@Pd(0)** nanoparticles remained as homogeneous solutions (Figure S9) when stored at room temperature in a glovebox. This observation indicates the importance of amphiphilic polymers in the stabilization of the Pd(0) in water. Elemental analysis was performed using energy-dispersive X-ray spectroscopy (EDS) experiments in combination with HAADF-STEM for all **P@Pd(0)** and the results confirmed the presence of palladium in all of them (Figure S10).

**Figure 1:**
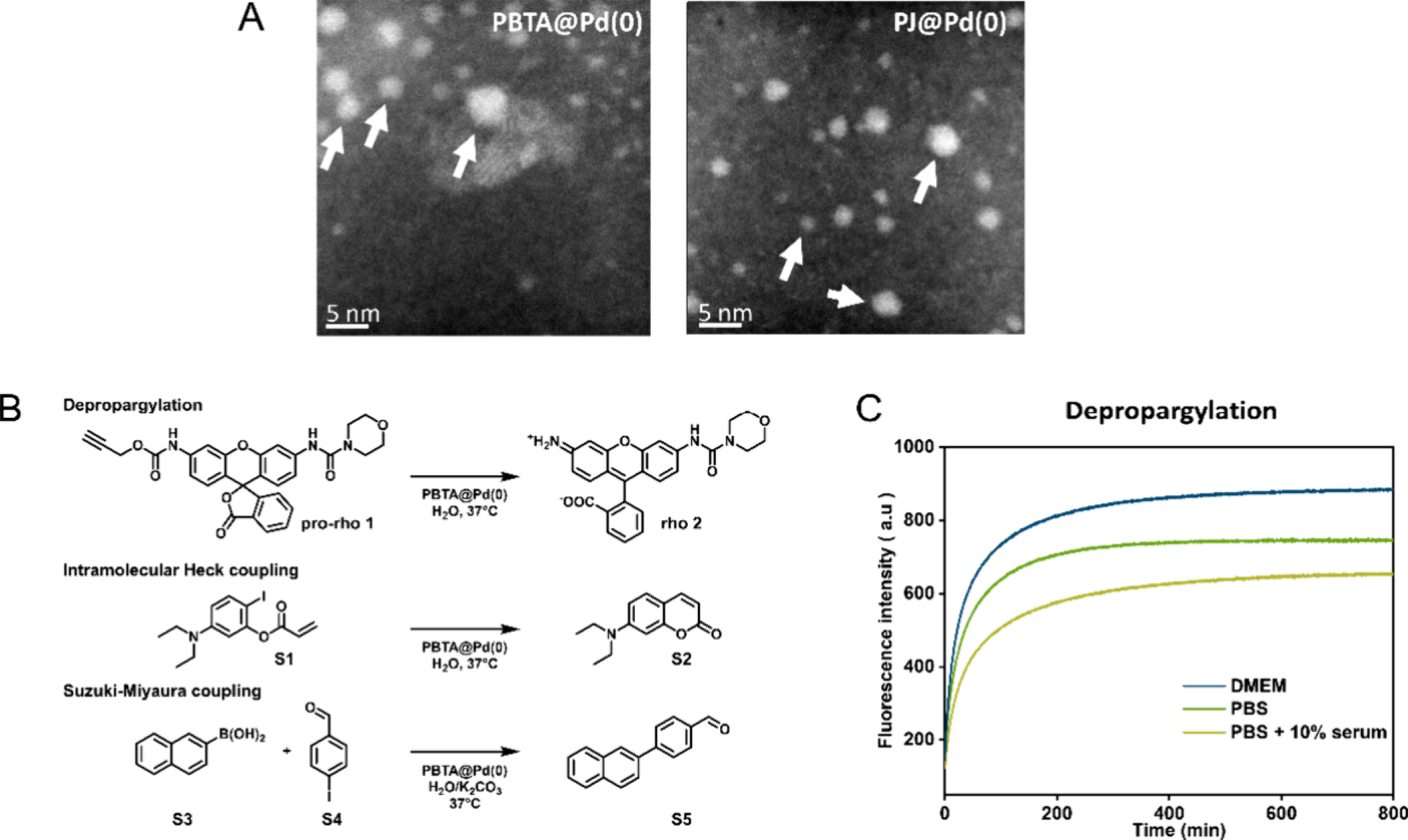
(A) Representative HAADF-STEM images of **P@Pd(0)**. (B) Three different types of chemical reactions with **PBTA@Pd(0)** performed at 37 °C in H_2_O. (C) Fluorescence kinetic profiles of **pro-rho 1** activation using **PBTA@Pd(0)** in PBS, DMEM, and PBS supplemented with 10% fetal bovine serum. The formation of **rho 2** was monitored at λ_ex_ = 485 nm and λ_em_ = 520 nm. Reaction conditions: [**1**] = 30 μM, [Pd(0)] = 30 μM, [**P**] ∼ 0.142 mg/mL, *T* = 37 °C.

In order to test the catalytic efficiency of the system, **PBTA@Pd(0)** was selected to perform depropargylation, Heck, and Suzuki-coupling reactions in water (Figure 1B). In all cases quantitative conversion to the corresponding products was observed (see SI, Figures S11–13). The efficiency of **PBTA@Pd(0)** to function in more complex media was explored in more detail for the depropargylation reactions on *N*-propargyl-protected rhodamine **pro-rho** (**1**). The reactions were performed in PBS, DMEM, and PBS supplemented with 10% fetal bovine serum (FBS). The formation of fluorescent **rho** (**2**) was followed by fluorescence spectroscopy over time and the conversion was monitored using HPLC-UV. In all cases, fast kinetics was observed despite the complexity of the medium, and near quantitative conversion was observed after 24 h using HPLC-UV (Figure 1C and Figure S14). A comparison between the catalytic efficiency of **PBTA@Pd(II)** and **PBTA@Pd(0)** was conducted by the depropargylation of **pro-rho** (**1**) in DMEM medium (Figure S15), which clearly indicated that the reduction of Pd(II) to Pd(0) resulted in faster kinetics in pro-dye activation. Bare Pd(0) NPs did not activate **pro-rho** (**1**) under the same reaction conditions, highlighting the importance of amphiphilic polymers in stabilizing the Pd(0) NPs and solubilizing the substrates.

To perform the catalysis experiments in the presence of cells, the biocompatibility of **pro-rho** (**1**) and **P@Pd(0)** with HepG2 cells, a widely used cancer cell line for toxicity testing, was studied. **P@Pd(0)** (Pd concentration: 60 μM) and **pro-rho** (**1**) (25 μM) were separately incubated with HepG2 cells for 48 h. Evaluation of cell viability using the CCK-8 assay (Figure S16) shows that the catalytic nanoparticles and **pro-rho** (**1**) do not exert toxicity under this condition, with cell viability remaining high around 95%. This indicated that developed Pd(0) based polymeric nanoparticles and pro-rhodamine have good biocompatibility.

Next, the catalysts **P@Pd(0)** and **pro-rho** (**1**) were simultaneously incubated with HepG2 cells for 2 h in a full cell culture medium (DMEM supplemented with 10% fetal bovine serum), and the catalytic activity was monitored by fluorescence spectroscopy over time (Figure 2A). The TPP ligand-based Pd(0) polymeric nanoparticles, **PBTA@Pd(0)**, **PJ@Pd(0)** and **PCOOH@Pd(0)**, exhibited considerably faster kinetics compared to **Pcontrol@Pd(0)**, which indicates that the presence of TPP ligands plays an important role in the catalytic efficiency or stabilization of the Pd(0) NPs. This is also corroborated by the better catalytic performance of **PJ@Pd(0)** and **PCOOH@Pd(0)**, which have the highest amount of TPP ligands (20%), as compared to **PBTA@Pd(0)** which contains only 10% TPP ligand (Figure 2A). Upon pro-rhodamine deprotection, the formed fluorescent product can be internalized by HepG2 cells and visualized via confocal microscopy. Therefore, this probe serves as a surrogate for extracellular prodrug activation and subsequent cell entry. To ensure sufficient internalization of the product, **P@Pd(0)** and **pro-rho** (**1**) were coincubated with HepG2 cells for 36 h. As can be seen in Figure 2B, **pro-rho** (**1**) barely shows fluorescence in cells, suggesting the stability of the propargyl protection group in the biological media. In contrast, in the presence of **P@Pd(0)**, fluorescent **rho** (**2**) was visible in cells, albeit with different emission intensities. **PJ@Pd(0)** and **PCOOH@Pd(0)** exhibit the highest intensity as quantified in Figure 2C, followed by **PBTA@Pd(0)** and **Pcontrol@Pd(0)**, which is consistent with the results of the kinetic profiles shown in Figure 2A. Intracellular catalysis for **pro-rho** (**1**) activation was also explored using **PCOOH@Pd(0)** which possesses Nile Red that can visualize the localization of nanoparticles inside cells. The lack of fluorescence in the **rho (2)** channel shown in confocal images (Figure S17) suggests that extracellular catalysis dominates the **pro-rho** (**1**) activation in the presence of cells. Overall, the pro-dye activation study shows that Pd(0) based NPs are efficient catalysts in the biological environment, and the TPP ligand plays an important role in their catalytic efficiency.

**Figure 2:**
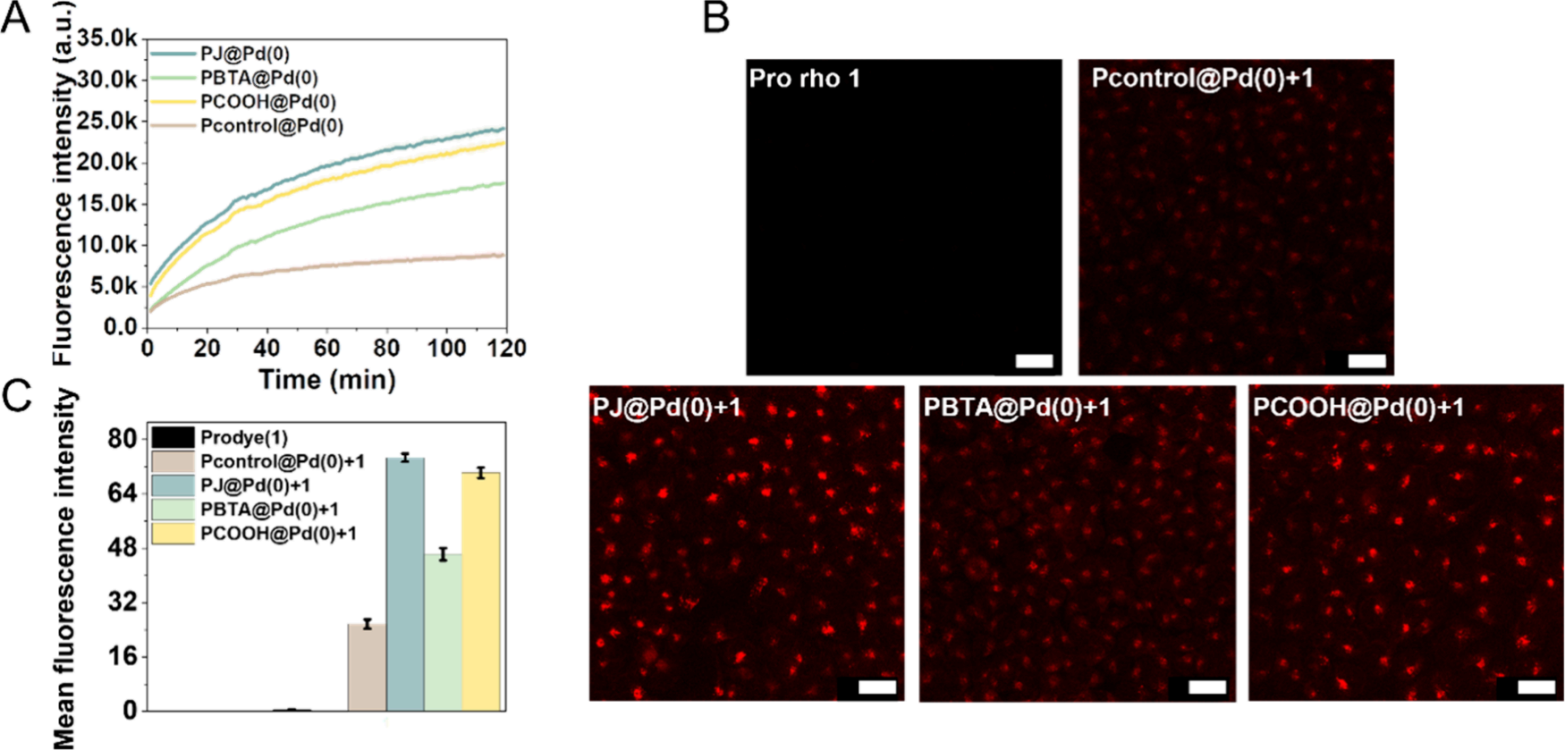
Deprotection of propargyl-protected rhodamine (**pro-rho** (**1**)) by **P@Pd(0)** in the presence of HepG2 cells. (A) Fluorescence kinetic profiles of **pro-rho** (**1**) (25 μM) activation using **P@Pd(0)** ([**Pd(0)**]: 60 μM), where the formation of rhodamine was monitored at λ_ex_ = 485 nm and λ_em_ = 520 nm at 37 °C. (B) Confocal images of fluorescent rhodamine in HepG2 cells. Scale bar: 20 μm. (C) Fluorescence intensity quantification of **rho** (**2**) derived from (B).

Encouraged by the above results, their potential for prodrug activation to induce cancer cell death was investigated next. Previously reported pro-drugs based on 5-fluorouracil (5FU) and doxorubicin protected with a propargyl group were explored.^4,5,29^ Three types of prodrugs, **pro-5FU** (**3**), **pro-Di5FU** (**5**), and **pro-dox** (**6**), were chosen for studying the catalytic potential of Pd(0)-SCPNs to induce cell death. It was previously reported that all three prodrugs are biologically inert in the absence of catalytic SCPNs.^4,5,29^ In addition, **pro-5FU** (**3**) required more than 6 h to achieve 50% conversion while **pro-Di5FU** (**5**) took less than 1 h for full conversion under the same reaction conditions in PBS, albeit using large Pd-functionalized microdevices.^4^

For pro-drug activation studies, we first evaluated the activation of **pro-5FU** (**3**) (100 μM) with **P@Pd(0)** by incubating both together with HepG2 cells for 48 h. **Pro-5FU** (**3**) and **P@Pd(0)** were incubated separately as the control experiment. Surprisingly, only **PJ@Pd(0)** in combination with **pro-5FU** (**3**) caused a prominent decrease in cell viability at a concentration of 80 μM or above (Figure 3). This highlights the *in situ* generation of 5FU in the presence of **PJ@Pd(0)**. However, the toxigenic effect created by the **pro-5FU**/catalyst combination was not observed for **PCOOH@Pd(0)**, **PBTA@Pd(0)**, and **Pcontrol@Pd(0)**. This could be attributed to the hydrophilic nature of the polymer **PJ**, which may facilitate the easy access of the hydrophilic prodrug **pro-5FU** to SCPNs, allowing for its conversion. In addition, it is found that **PJ@Pd(0)** possesses high catalyst stability *in vitro* (results shown below). The same trend was observed in the case of **pro-Di5FU** (**5**) activation, where a significant decrease in cell viability was only observed in case **PJ@Pd(0)** (Figure S18).

**Figure 3:**
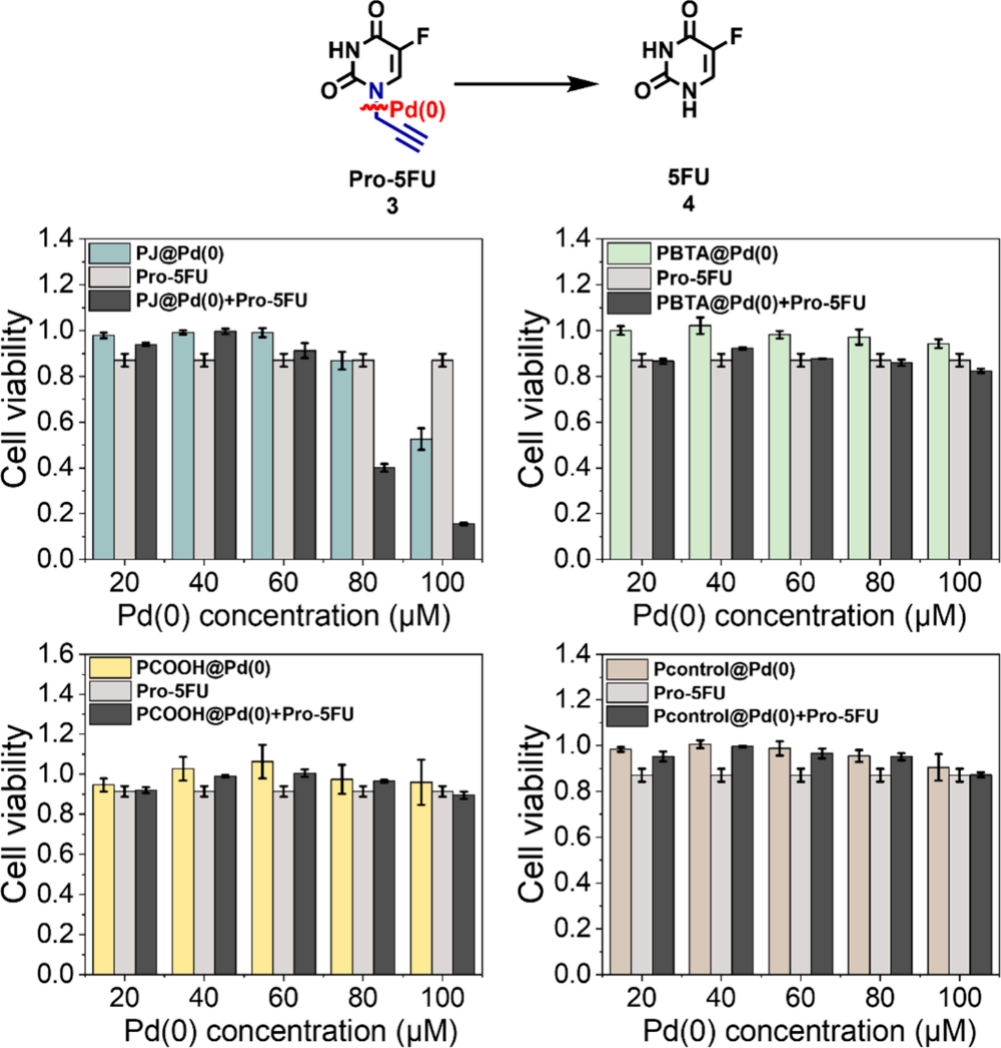
Activation of **pro-5FU** (**3**) (100 μM) by Pd(0) based SCPNs in the presence of HepG2 cells (incubation time: 48 h).

Significantly different from 5FU, doxorubicin is reported to induce rapid apoptosis at low concentrations.^30^ The incubation of doxorubicin (10 μM) with HepG2 cells for 48 h causes a remarkable reduction of cell viability to around 20% (Figure S19). The catalytic efficiency of **P@Pd(0)** in a range of concentrations for **pro-dox** (**6**) activation was next explored. **Pro-dox** (**6**) (10 μM) and **P@Pd(0)** were incubated in varying concentrations separately with HepG2 cells as control experiments, and mixing the two in the presence of cells was for studying **pro-dox** (**6**) activation. As can be seen in Figure 4, neither **P@Pd(0)** nor **pro-dox** (**6**) exerted toxicity to cells, but when the two were mixed, significant cell death was observed (around 20% cell viability), even at the lowest Pd(0) concentration of 20 μM, and regardless of the microstructure of the polymers. This indicates that the activation of **pro-dox** (**6**) is extremely efficient by Pd(0) based SCPNs. For comparison, the catalytic efficiency of **Pd(II)** based SCPNs for **pro-dox** (**6**) activation (Figure S20) was studied. The results show that only TPP ligand-based catalytic systems, including **PJ@Pd(II)**, **PBTA@Pd(II)**, and **PCOOH@Pd(II)**, were able to convert **pro-dox** (**6**) at a low concentration of catalysts, while **Pcontrol@Pd(II)** requires a much higher Pd(II) concentration (100 μM) to cause HepG2 cell death upon deprotection of **pro-dox** (**6**).

**Figure 4:**
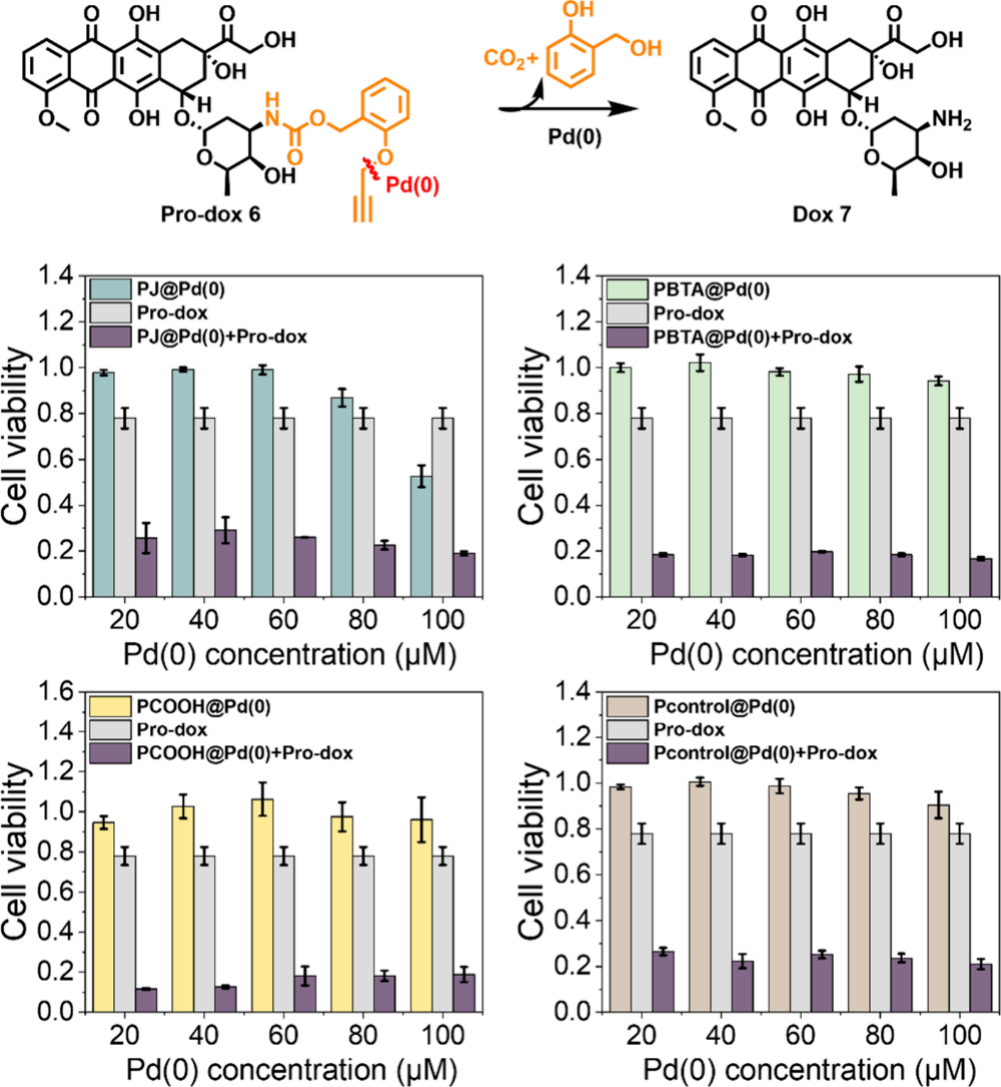
Activation of **pro-dox** (**6**) (10 μM) by Pd(0) based SCPNs in the presence of HepG2 cells (incubation time: 48 h).

In order to function in biological environments, catalysts should show high stability to metabolism over long time periods. To study how the microstructures of the polymers affect the stability of the **P@Pd(0)** catalyst in the presence of cells, the effectiveness of the nanodevices to activate **pro-dox** (**6**) was evaluated after different times of preincubation in HepG2 culture. Of note, HepG2 cells are widely used as hepatocyte models due to their high level of differentiation. They display many of the genotypic features of normal liver cells,^31,32^ thereby serving as an excellent model to challenge the stability of the nanocatalysts. Hereto, **P@Pd(0)** (80 μM) were incubated with HepG2 cells for 0.5 h, 1 h, 1.5 h, 2 h, and 3.5 h prior to adding **pro-dox** (**6**) to assess how long the catalyst remains active in the biological environment. If the catalysts are stable for a certain period in the cell culture media, it is expected that drug formation will induce significant cell death after adding **pro-dox** (**6**). Otherwise, cell viability will remain high, thereby indicating the deactivation of catalysts. As a control, **P@Pd(0)** and **pro-dox** (**6**) were mixed prior to adding them to cells, to obtain the highest toxigenic effect, which corresponds to *t* = 0 h incubation.

Figure 5 shows the cell viability after incubating the cells for the set time periods with the catalyst **P@Pd(0)** prior to adding **pro-dox** (**6**), compared to the simultaneous addition of the catalysts and **pro-dox** (**6**) (*t* = 0 h). As previously seen, the cell viability decreased significantly at *t* = 0 h by combining the catalysts and **pro-dox** (**6**) before addition to cells, indicating the highest toxigenic effect based on **P@Pd(0)** systems (Figure 5). After 3.5 h of catalyst preincubation with cells, **PJ@Pd(0)** and **PCOOH@Pd(0)** showed slightly reduced, but still satisfactory, catalytic activity when adding **pro-dox** (**6**). On the contrary, **PBTA@Pd(0)** showed clear evidence of deactivation, indicated by the high cell viability (80%) upon deprotection of **pro-dox** (**6**). This suggests that a sufficient amount of TPP ligand (20%) is beneficial for stabilizing the Pd catalysts for a longer period of time.

**Figure 5:**
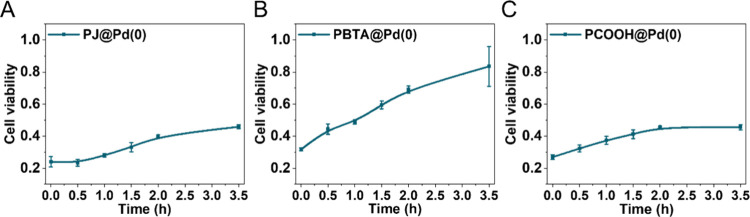
Incubation of **P@Pd(0)** ([Pd(0)]: 80 μM) with HepG2 cells for 0 h, 0.5 h, 1 h, 1.5 h, 2 h, and 3.5 h prior to adding **pro-dox** (**6**) (10 μM) for the deactivation study. The time point *t* = 0 represents the cell viability observed after adding **P@Pd(0)** and **pro-dox** (**6**) simultaneously to cells to obtain the highest toxigenic effect.

In order to compare the extent of deactivation, the corresponding Pd(II) catalysts **P@Pd(II)** were also evaluated for their stability, as they are known for faster deactivation by nucleophiles. When incubated with cells for 3.5 h followed by addition of **pro-dox** (**6**), the Pd(II) loaded SCPNs showed high cell viability for **PBTA@Pd(II)** and **PJ@Pd(II)**, suggesting complete deactivation (Figure S21). In contrast, **PCOOH@Pd(II)** was still active after 3.5 h of incubation with cells, as indicated by a low cell viability (40%) upon deprotection of **pro-dox** (**6**) (Figure S21). This effect can be attributed to the presence of the −COOH group which likely precluded the leaching of Pd(II) through the electrostatic interactions, a key factor in maintaining the active sites inside the catalyst. The stability study of Pd(II) and Pd(0) based SCPNs suggests that **P@Pd(0)** generally remains active for a longer period time compared to **P@Pd(II)** in the presence of cells. The results of the Pd stability study highlight that screening all potential bioorthogonal catalysts for their stability *in vitro* will help in finding suitable candidates for *in vivo* studies.

In this study, toxicity induced by the activation of prodrugs was observed at low Pd(0) concentrations. Especially for **pro-dox** activation, a mere 20 μM of Pd(0) proved to be effective in activating the potent anticancer drug doxorubicin, and significant cell death was observed. Notably, as compared to other bioorthogonal catalysts where a 5-day incubation period is required for **pro-5FU** activation, our system accomplished catalysis within 48 h in the presence of cells.^24^ It is known that HepG2 cell lines are very resistant and are tough targets for 5FU to exert its function,^31,32^ explaining why the activation of 5FU was less prone to lead to significant cell death. The resistance of HepG2 toward 5FU also helped to unveil differences in catalytic activity resulting from the different polymer microstructures in **P@Pd(0)**. **PJ@Pd(0)** activated **pro-5FU**, **pro-Di5FU**, and **pro-dox**, leading to a significant decrease in cell viability, making it a promising candidate for further *in vivo* studies.

In conclusion, we successfully developed highly effective Pd(0) based amphiphilic polymeric nanoparticles by utilizing CO as a mild reductant to reduce Pd(II) in the presence of the amphiphilic polymers. Pd(0) loaded polymeric nanoparticles exhibited fast kinetics in prodye activation and displayed great efficacy in converting prodrugs *in vitro*. By modifying the polymer microstructure, a combination of 20% TPP ligand and 80% Jeffamine M-1000 group was identified as the highly efficient catalyst, capable of converting pro-drugs of 5FU and doxorubicin into their active forms and inducing HepG2 cell death, while all polymer designs are equally suitable for pro-dox activation by applying the Pd(0) catalyst. The stability studies revealed that a higher ratio of TPP ligand can better stabilize catalysts and help to retain the catalytic activity over time in biological environments. These findings provide crucial insights for the rational design of Pd-based amphiphilic polymers to bridge the gap between *in vitro* and *in vivo* experiments, thereby improving the overall success rate.

## Supporting Information Available

Additional experimental details, materials, and methods, synthesis and characterization details of polymers, formulation of catalytic nanoparticles, cell viability studies, and prodrug activation in the presence of cells (PDF)
