# Supplementary material for: Enhanced Efficiency of Pd(0)-Based Single Chain Polymeric Nanoparticles for in Vitro Prodrug Activation by Modulating the Polymer’s Microstructure
Source: Nano Lett. 2024 Feb 12;24(7):2242–9. doi: 10.1021/acs.nanolett.3c04466 (PMC10885199; doi:10.1021/acs.nanolett.3c04466)
Supplement: Supplementary file 1 — nl3c04466_si_001.pdf [file nl3c04466_si_001.pdf]

Enhanced Efficiency of Pd(0)-based Single Chain Polymeric Nanoparticles for In Vitro Prodrug Activation by Modulating the Polymer's Microstructure

Linlin Deng[1,2]‡, Anjana Sathyan[1,2]‡, Catherine Adam[3], Asier Unciti-Broceta[3], Víctor Sebastian[4,5,6,7] and Anja R. A. Palmans[1,2]*

[1] Laboratory for Macromolecular and Organic Chemistry, Department of Chemical Engineering and Chemistry, Eindhoven University of Technology, P.O. Box 513, 5600 MB Eindhoven, The Netherlands.
[2] Institute for Complex Molecular Systems, Eindhoven University of Technology, P.O. Box 513, 5600 MB Eindhoven, The Netherlands.
[3] Edinburgh Cancer Research, Cancer Research UK Scotland Centre, Institute of Genetics and Cancer, University of Edinburgh, Crewe Road South, Edinburgh, EH4 2XR, UK.
[4] Instituto de Nanociencia y Materiales de Aragón (INMA), CSIC-Universidad de Zaragoza, Zaragoza 50009, Spain.
[5] Department of Chemical and Environmental Engineering Universidad de Zaragoza Campus Rio Ebro, 50018 Zaragoza, Spain.
[6] Laboratorio de Microscopías Avanzadas, Universidad de Zaragoza, 50018 Zaragoza, Spain.
[7] Networking Research Center on Bioengineering, Biomaterials and Nanomedicine (CIBER-BBN), 28029 Madrid, Spain.
* Corresponding author: a.palmans@tue.nl
‡ These authors contributed equally

1. Materials and Methods

All chemicals were purchased either from Merck or TCI chemicals. Deuterated solvents were purchased from Cambridge isotope laboratories. The dialysis membrane was regenerated cellulose tubing purchased from Spectra/Por® with a molecular weight cut-off of 6-8 kDa. All solvents were purchased from Biosolve. Gibco DMEM (glucose concentration 1 g/L, with sodium pyruvate, without L-glutamine, without phenol red) and MEM medium were purchased from Fischer scientific. Dialysis of triphenylphosphine (TPP) functionalised polymer was done in degassed solvents in a wide screw-capped 1 L container, keeping it tightly closed. Dialysis solvents were refreshed regularly with new degassed solvents to prevent oxidation of phosphines. Chloroform used for triphenylphosphine polymers' complexation was dried using Mbraun solvent purification system (MB-SPS 800) and was degassed thoroughly by six freeze-pump thaw cycles. All the flasks and needles used for TPP polymers were pre-dried in the oven at 135 °C overnight, and experiments were performed under an argon atmosphere. Automated column chromatography was performed on Grace Reveleris X2 Flash Chromatography System using Flashpure BUCHI prepacked silica columns. The polymers were synthesized according to previously reported protocol in our group. **p-PFPA$_{180}$** and **p-PFPA$_{200}$** were synthesized according to previously reported protocol.[1] Polymer **PBTA** and **PJ** were same polymers as reported before.[2] **PCOOH** and **Pcontrol** were synthesized following the same protocol.[2] Nile red amine was synthesized according to previously reported protocol.[3] All pro-drugs and the pro-dye were synthesized following the previously reported protocol.[2,4–6]

Fluorescence measurements were performed on an Agilent Cary Eclipse fluorescence spectrophotometer using 1 cm × 1 cm pathlength quartz cuvettes. Liquid chromatography - UV was performed using Shimadzu UFLC-XR with PDA detector with water + 0.1% formic acid and ACN + 0.1% formic acid as eluents on Kinetex column C18 5 mm EVO 100 Å. HPLC Method for Heck coupling, Suzuki-Miyayura coupling and depropargylation reactions: eluent A: water (0.1% formic acid); eluent B: acetonitrile (0.1% formic acid); and A/B = 90:10 isocratic 2 min, 90:10 to 0:100 in 2 min, isocratic 2 min, 0:100 to 90:0 in 2 min, and isocratic 2.0 min (flow = 0.2 mL/min). High-Performance Liquid Chromatography – HPLC-UV/MS was performed on a SHIMADZU Nexera-I LC-2040C 3D coupled with LC-MS 2020 for detection. Method 2 for pro-5FU: A/B = 95:5 isocratic 15 min on Hypercarb column.

DMF-SEC measurements of functionalised polymers were performed using PL-GPC-50 plus (Varian Inc. Company) equipped with a refractive index detector. DMF with 10 mM LiBr was used as eluent at a flow rate of 1 mL min^{-1} on the Shodex GPC-KD-804 column at 50 °C. Exclusion limit = 100.000 Da, 0.8 cm i.d. × 300 mm calibrated using poly (ethylene oxide) from polymer laboratories. Dynamic light scattering experiments were performed using Malvern Zetasizer with 830 nm laser and an angle of scattering 90°. For cell experiments, analysis of the microplate was performed using a Tecan MC-SPARK. Confocal microscopy images of HeLa cells were obtained with a Leica SP5 confocal microscope with a HyD2 detector. Identical conditions were followed throughout all measurements. ImageJ was used for processing images.

High angle annular dark field scanning transmission electron imaging (HAADF-STEM) with energy-dispersive X-ray spectroscopy (EDS) analysis was performed at the LMA-ELCEMI ICTs with a field emission gun microscope (Analytical XFEG FEI Titan, 300 kV) equipped with Cs-probe allowing 0.09 nm mean size electron probe formation (CEOS).

2. Experimental Procedures

Table S1: Overview of the copolymer composition (a-f), degree of polymerisation (n), molecular weight ($M_{n,SEC}$) and molar mass dispersity (Đ) of **PBTA-Pcontrol** before complexation to Pd(II). Dynamic light scattering results of the polymers complexed to Pd(II) and after CO reduction to Pd(0) in H_2O.

Polymer	a	b	c	d	e	f	n	Đ	$M_{n,SEC}$ (kD)	R_H (nm) P@Pd(II)	R_H (nm) P@Pd(0)
**PBTA**	8	68	4	20	-	-	214	1.18	46.8	6.6 ± 0.6	8.1 ± 0.9 (43±24)[a]
**PJ**	18	82	-	-	-	-	214	1.34	55.4	5.7 ±1.7	6.2 ± 0.3 (15 ± 1)[b]
**PCOOH**	20	44	-	-	35	1	180	1.26	36.5	6.6 ± 1.5	4.6 ± 1.0 (13 ± 2)[c]
**Pcontrol**	-	70	-	30	-	-	200	1.16	24.4	5.9 ± 0.9	8.8 ± 0.7 (67 ± 7)[d]

a-f were determined by ^{19}F NMR. $\mathbf{M_n}$ and **Đ** were measured by SEC in DMF with 10 mM LiBr, relative to poly(ethylene oxide) standards for polymers before complexation to Pd(II). $\mathbf{R_H}$ was determined by dynamic light scattering experiments [**P**] = 1 mg/mL, [Pd(II)] = [Pd(0)] = 210 µM in H_2O. In all cases, $\mathbf{R_H}$ was determined after the filtration of particles using a 100 nm PVDF filter. The $\mathbf{R_H}$ was determined from the volume plot of the DLS results, the values in the bracket correspond to a small fraction of larger aggregates present as follows [a]3%, [b]4%, [c] 2%, [d]3%.

Synthesis of **PCOOH**: **p-PFPA$_{180}$** (100 mg, 1 eq, 0,0023 mmol) was dissolved in dry and degassed DMF in a Schlenk flask kept in a preheated oil bath at 50 °C. To this solution Nile red amine[15] (3 mg, 3 eq, 0.0069) was added and stirred overnight, the reaction was monitored using ^{19}F NMR by comparing the peaks of free pentafluorophenol with those in the polymer backbone. The incorporation of Nile red amine was found to be 1% after overnight stirring. To this solution, TPP ligand N-(6-aminohexyl)-4-(diphenylphosphaneyl) benzamide (33 mg, 36 eq, 0,082 mmol) was added and stirred overnight. Followingly, 100 µL triethylamine (not dried) was added to the reaction mixture to trigger hydrolysis of the poly-(pentafluorophenol acrylate) backbone. After monitoring the amount of displaced pentafluorophenol using ^{19}F NMR, Jeffamine® M-1000 (414 mg, 180 eq, 0.41 mmol) was added. The reaction mixture was then left overnight under argon and the completion of the reaction was again monitored using ^{19}F NMR. Then, the reaction mixture was purified by dialysis (1 x 1 L methanol, 2 x 1 L THF) for 3 days in a tightly closed screw-capped container. Degassed solvents were used for dialysis which was refreshed as frequently as possible (> 6 h time gap) to prevent oxidation of triphenylphosphine. After dialysis, the THF volume was reduced to ~ 3 mL using a rotary evaporator and the polymer was precipitated into ice-cold pentane (800 mL). The precipitated polymer was washed again with ice-cold pentane, dried under argon flow and was then transferred to a small glass vial. The polymer was then dried under vacuum overnight at 50 °C to yield a bright pink solid and was stored at -19 °C wrapped with aluminium foil. Yield: 42 mg. $M_{n,\ SEC\text{-}DMF}$ = 36.5 kD. Đ = 1.26.

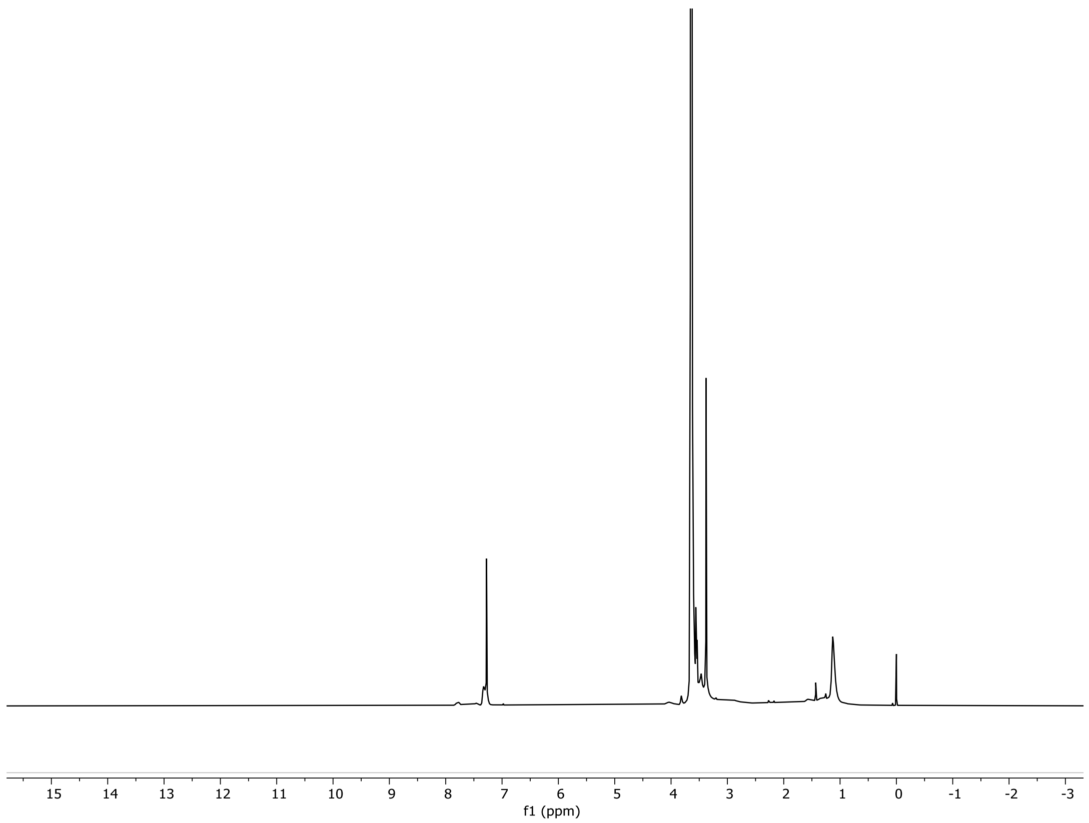

Figure S1: ^{1}H NMR of polymer **PCOOH** in $CDCl_3$.

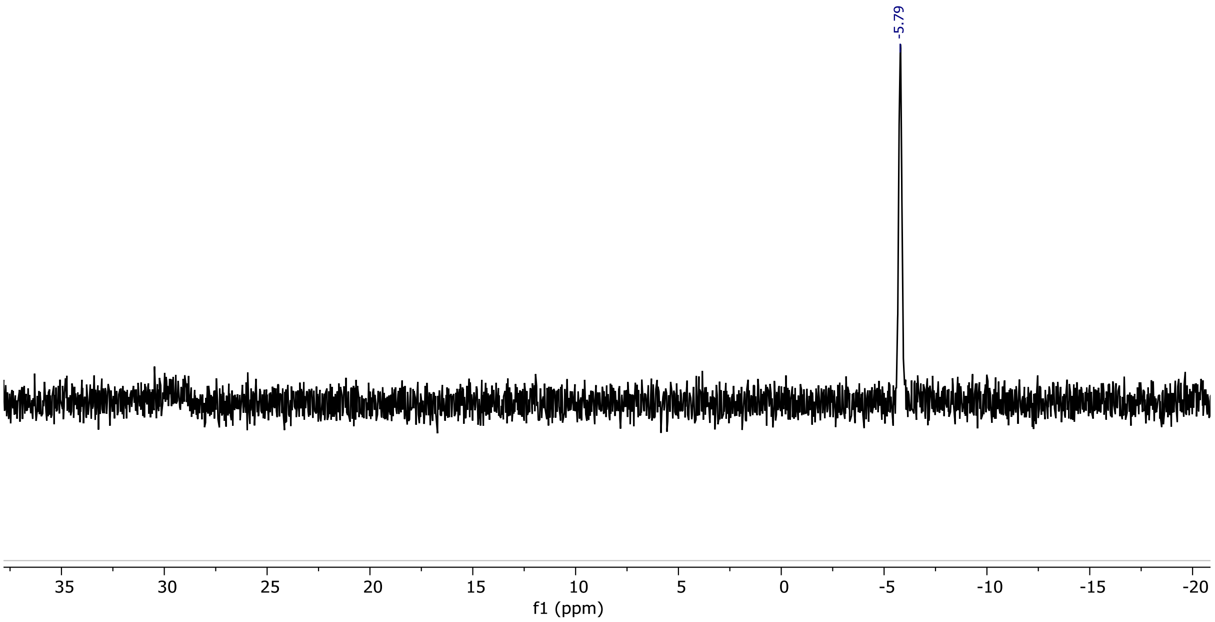

Figure S2: ^{31}P NMR of polymer **PCOOH** in $CDCl_3$. The peak at -5 ppm belongs to triphenylphosphine (TPP) ligands attached to the polymer backbone. Slight oxidation of TPP ligands was also observed as from the peak at 29 ppm indicating the presence of triphenylphosphine oxide.

Synthesis of **Pcontrol**: Synthesis was performed similar to above protocol with varying ligand ratio as follows: **p-PFPA$_{200}$** (100 mg, 1 eq, 0,0020 mmol), dodecyl amine (22 mg, 60 eq, 0.12 eq, Jeffamine® M-1000 (360 mg, 180 eq, 0.36 mmol). Dialysis was performed same as

PCOOH but degassing was not performed. The polymer was dried under vacuum overnight at 50 °C to yield a pale solid and was stored at -19 °C. $M_{theoretical}$ = 181 kD, $M_{n, SEC-DMF}$ = 24.4 kD, Đ = 1.16.

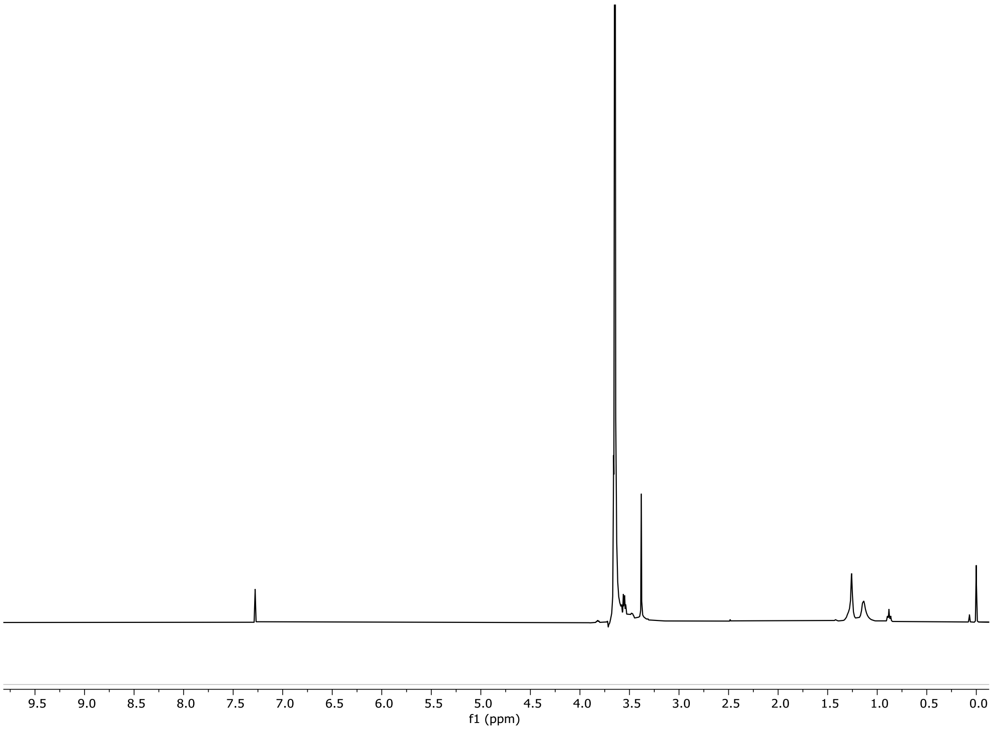

Figure S3: ^{1}H NMR of polymer **Pcontrol** in $CDCl_3$.

Preparation of **P@Pd(0):**

All polymers were formulated to nanoparticles by dissolving 10 mg of polymer **PBTA-Pcontrol** in 9979 µL degassed MilliQ water, followed by the addition of 21 µL of Pd(COD)Cl_2 stock solution (100 mM in DMSO) to reach a final concentration of 210 µM Pd(II) in 1 mg/mL polymer solution. Reduction of **P@Pd(II)** to **P@Pd(0)** was done using a gas-phase reduction process in a stainless steel Teflon lined autoclave. The resulting homogeneous solution was introduced into the autoclave and gently stirred with a magnetic stirrer. The autoclave was flushed under CO and pressurized to 6 bar. The autoclave was kept at 30 °C for 60 min. After the CO treatment, N_2 gas was introduced in the reaction vessel and the solution was further used. The samples after reduction were immediately transferred to a glove box under a nitrogen atmosphere. Samples kept under atmospheric conditions were found to show decreased reactivity after 1 week.

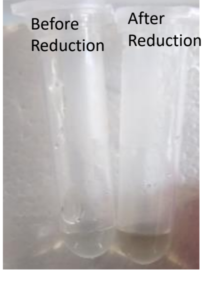

Figure S4: **P@Pd(II)** (before reduction) and **P@Pd(0)** (after reduction).

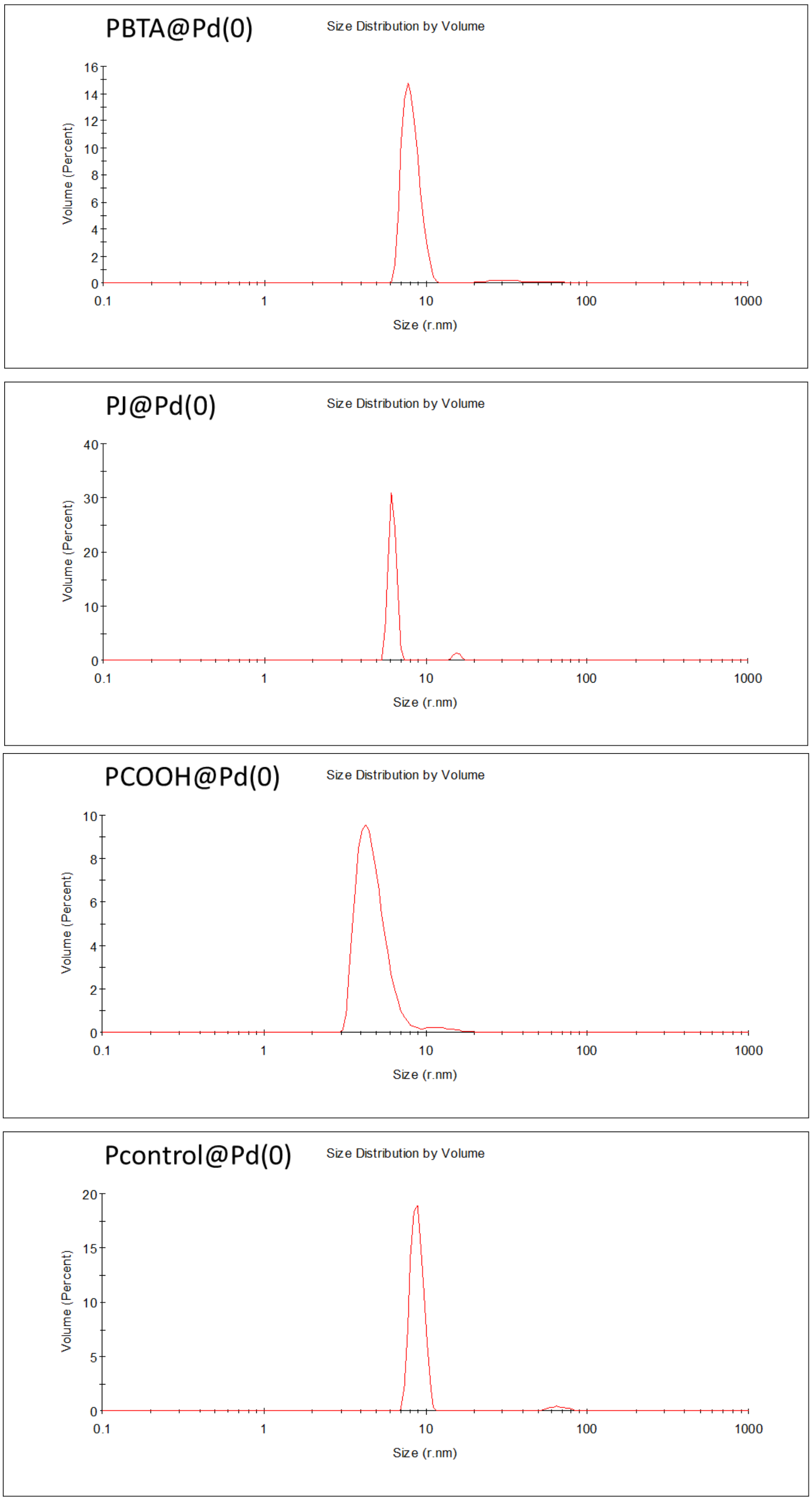

Figure S5: Dynamic light scattering measurements of **PBTA@Pd(0)**, **PJ@Pd(0)**, **PCOOH@Pd(0)** and **Pcontrol@Pd(0)**, [**P**] = 1 mg/mL, [Pd(0)] = 210 μM in H_2O.

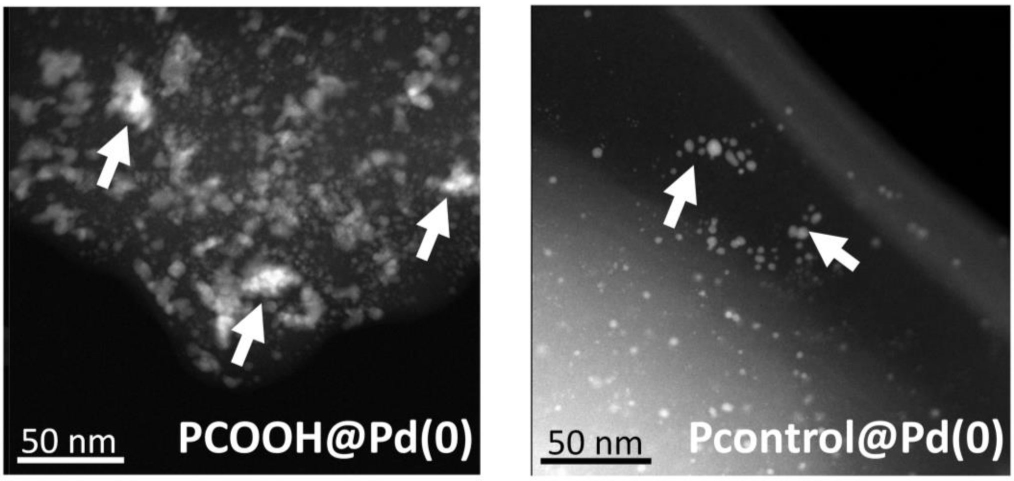

Figure S6: STEM-HAADF images of **PCOOH-Pcontrol@Pd(0)**

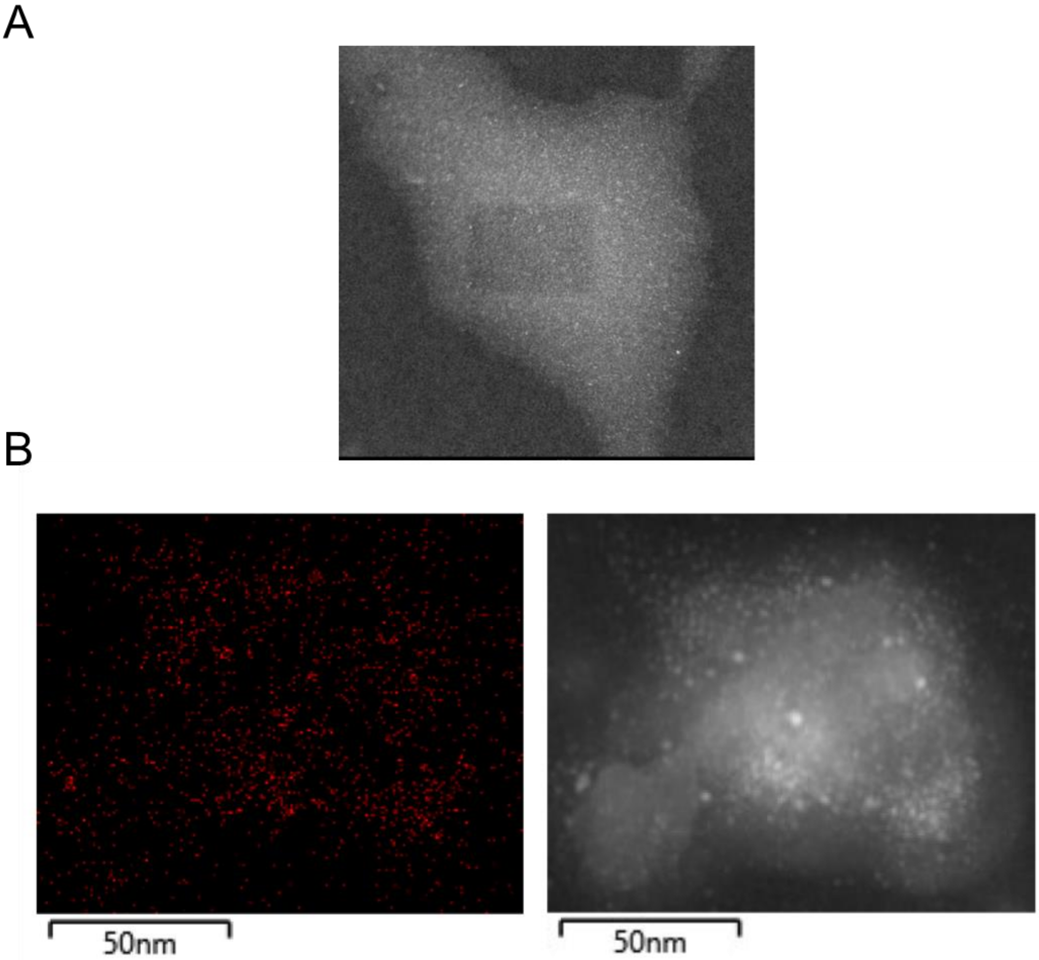

Figure S7: (A) Low magnification STEM-HAADF image of **P@Pd(0)**. The white dots refer to Pd nanoparticles since the HAADF detector has a Z contrast and Pd should have a brighter contrast than carbon. (B) A representative EDS mapping for the Pd element, where Pd co-localizes with the white dots.

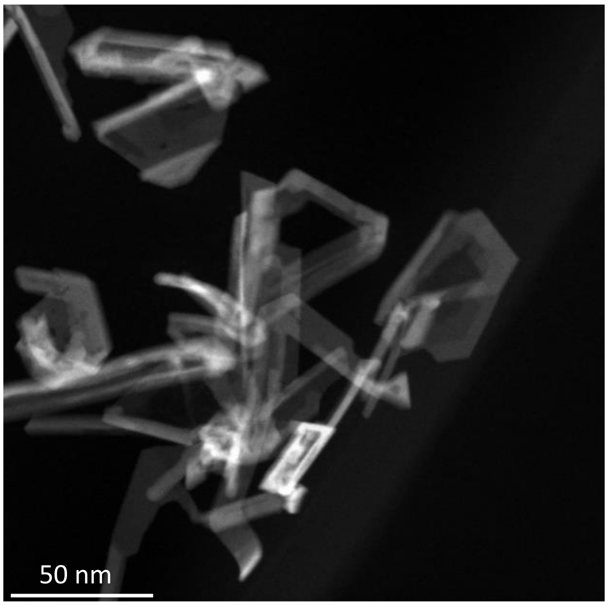

Figure S8: STEM-HAADF images of **Pd(0) NPs.**

A B

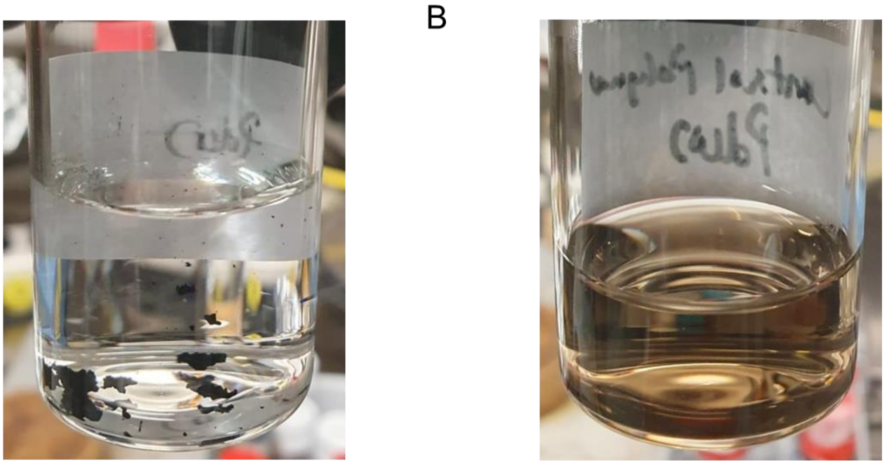

Figure S9: Image of the solutions containing Pd(0) NPs with and without polymers after two weeks. (A) **Pd(0) NPs** (no polymer present) (B) **Pcontrol@Pd(0) NPs**. [Pd] = 210 μM, H_2O, stored at RT under an inert atmosphere in a glove box.

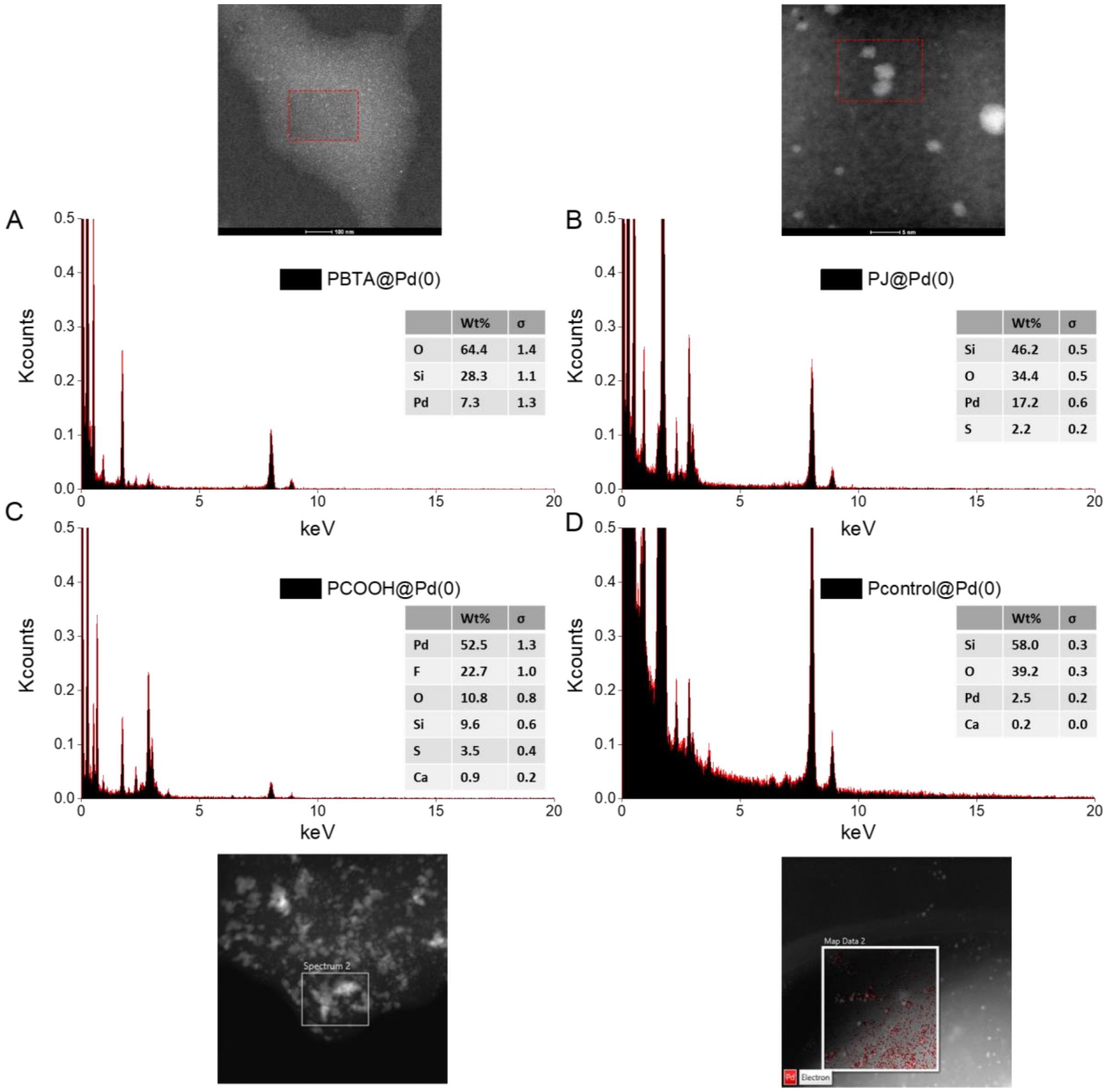

Figure S10: Elemental mapping using Energy Dispersive X-Ray Spectroscopy measurements on a mapped area of STEM-HAADF showing Pd wt% in the area of the sample **P@Pd(0)** measured.

General procedure for reactions in water :

Depropargylation reaction: Substrate stock solution (**1**) was prepared in DMSO at 30 mM concentration. **PBTA@Pd(0)** was at 210 μM Pd(0) concentration. The stock solution was diluted in 3 mL water in 10 mm fluorescence cuvette to a reach a final concentration of Pd(0) = 30 μM; [**1**] = 30 μM. Cuvettes were then transferred to fluorescence spectrophotometer at 37 °C with stirring and the reaction progress was monitored in real-time. Aliquots from the sample were taken at specified intervals and diluted with 50% ACN by volume which was then analysed using HPLC-UV.

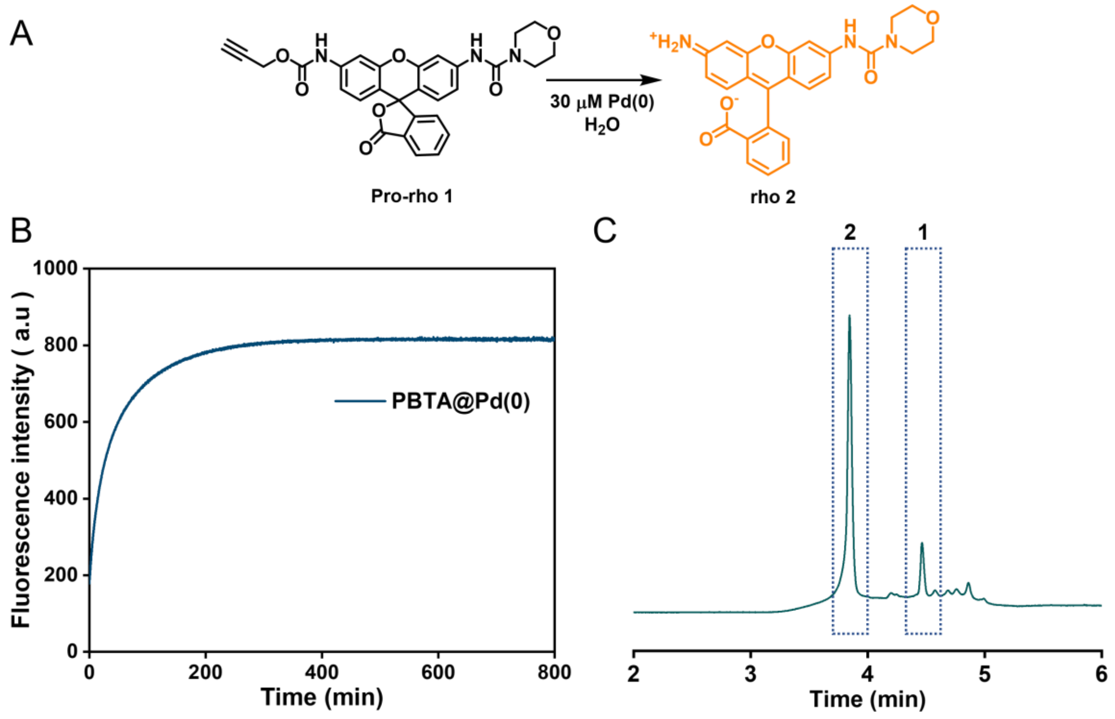

Figure S11: A) Activation of pro-rho **1** to rho **2** by depropargylation promoted by **PBTA@Pd(0)** in H_2O. B) Fluorescence kinetic profile of pro-rho **1** activation using **PBTA@Pd(0)** in H_2O where formation of rho **2** was monitored at λ_{ex} = 485 nm and λ_{em} = 520 nm, reaction conditions: [**1**] = 30 μM, [Pd(0)] = 30 μM, [**P**] ~ 0.142 mg/mL, T = 37 °C, in H_2O. C) HPLC-UV chromatogram of the reaction mixture after 24 h monitored at λ = 265 nm.

Heck coupling reaction: Substrate stock solution (**S1**) was prepared in DMSO at 30 mM concentration. **PBTA@Pd(0)** and **Pd(0)** stock solutions were at 210 μM Pd(0) concentration. Depending on each experiment, all stock solutions were diluted in 3 mL water in 10 mm fluorescence cuvette to a reach final concentration of Pd(0) = 30 μM; [**S1**] = 30 μM. Cuvettes were then transferred to fluorescence spectrophotometer at 37 °C with stirring and the reaction progress was monitored in real-time. Aliquots from the sample were taken at specified intervals and diluted with 50% ACN by volume which was then analysed using HPLC-UV.

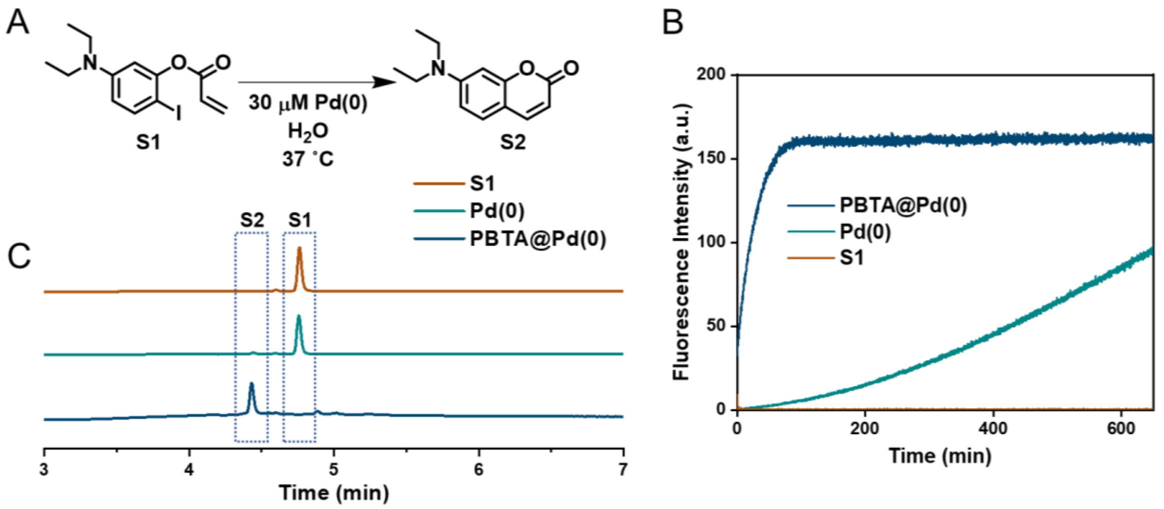

Figure S12: A) Formation of 7-diethylaminocoumarin **S2** from non-fluorescent precursor **S1** via intramolecular Heck reaction B) Fluorescence kinetic profile of Heck reaction promoted by **PBTA@Pd(0)** and **Pd(0)**, 7-diethylaminocoumarin **S2** monitored at λ_{ex} = 375 nm and λ_{em} = 500 nm. [**S1**] = 30 μM, [Pd(0)] = 30 μM, [**P**] ~ 0.142 mg/mL, T = 37 °C, in H_2O. C) HPLC-UV chromatogram from aliquots of the reaction mixture after 180 min.

Suzuki-Miyaura coupling reaction: Substrate stock solution **S3** was prepared in DMSO at 100 mM concentration, **S4** at 50 mM concentration, K_2CO_3 at 200 mM concentration. **PBTA@Pd(0)** and Pd(0) stock solutions were at 210 μM concentration. Depending on each experiment, all stock solutions were diluted in 3 mL water into a glass vial to a reach a final concentration of Pd(0) = 50 μM; [**S3**] = 100 μM; [**S4**] = 50 μM; [K_2CO_3] = 30 mM in water. The glass vial was then transferred to an oil bath at 37 °C with stirring and the reaction progress was monitored over time using HPLC-UV and MS. Aliquots from the sample were taken at specified intervals and diluted with 50% ACN by volume which was then analysed using HPLC-UV.

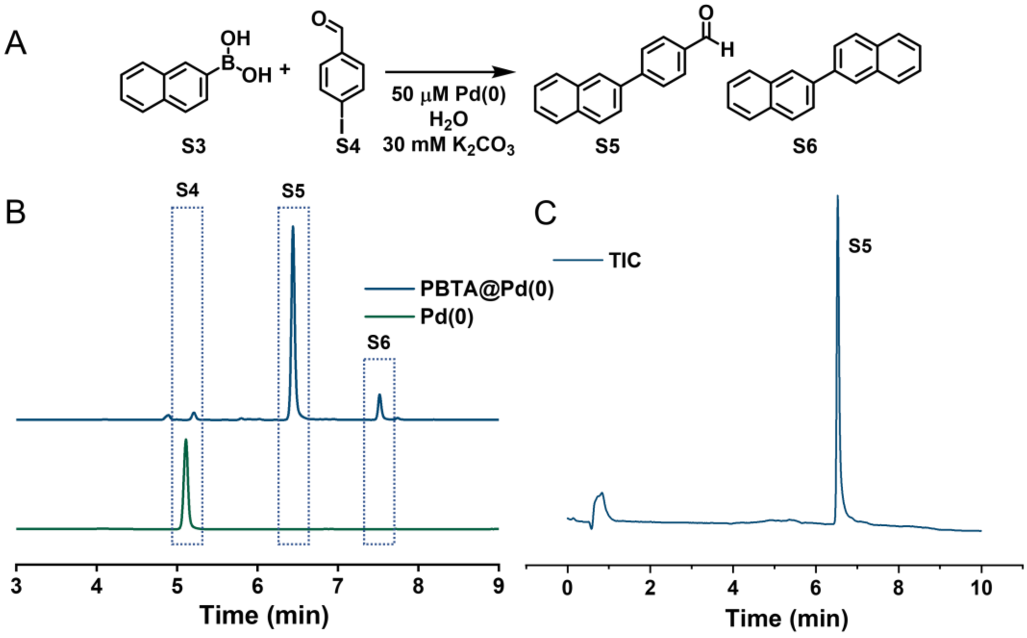

Figure S13: A) Suzuki-Miyaura coupling of naphthalene boronic acid **S3** and iodobenzaldehyde **S4** to form C-C coupled products B) HPLC-UV chromatogram of the reaction mixture using **PBTA@Pd(0)** and **Pd(0)** after 24 h C) HPLC-MS chromatogram of the same aliquot, from total ion current (TIC) channel showing mass $[M+H]^+ = 233$, corresponding to product **S5** (m/z = 232). Reaction conditions: [**S3**] = 100 μM, [**S4**] = 50 μM, [Pd(0)] = 50 μM, [**P**] ~ 0.238 mg/mL, T = 37 °C, in H_2O.

General procedure for reactions in complex media:

Pro-rho **1** activation in different media: Substrate stock solution (**1**) was prepared in DMSO at 30 mM concentration. **PBTA@Pd(0)** stock solution was prepared at 210 μM concentration. Depending on each experiment, all stock solutions were diluted in 3 mL water, PBS, DMEM or PBS supplemented with 10% FBS serum, in 10 mm fluorescence cuvette to a reach a final concentration of Pd(0) = 30 μM; [**1**] = 30 μM. Cuvettes were then transferred to fluorescence spectrophotometer at 37 °C with stirring and the reaction progress was monitored in real-time. Aliquots from the sample were taken at specified intervals and diluted with 50% ACN by volume which was then analysed using HPLC-UV.

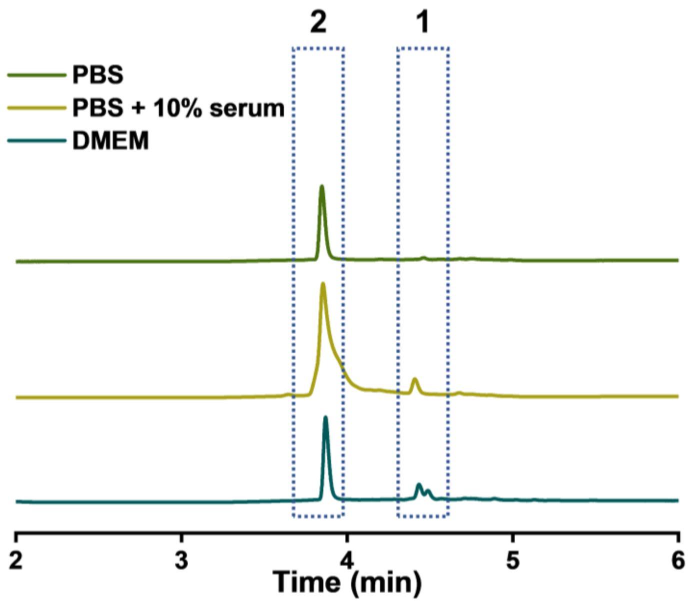

Figure S14: HPLC-UV chromatogram after depropargylation reaction on pro-rho **1** by **PBTA@Pd(0)** in PBS, PBS+10%FBS serum and DMEM after 24 h monitored at λ = 265 nm. Reaction conditions: [**1**] = 30 μM, [Pd(0)] = 30 μM, [**P**] ~ 0.142 mg/mL, T = 37 °C. B) HPLC-UV chromatogram of the corresponding reaction mixture.

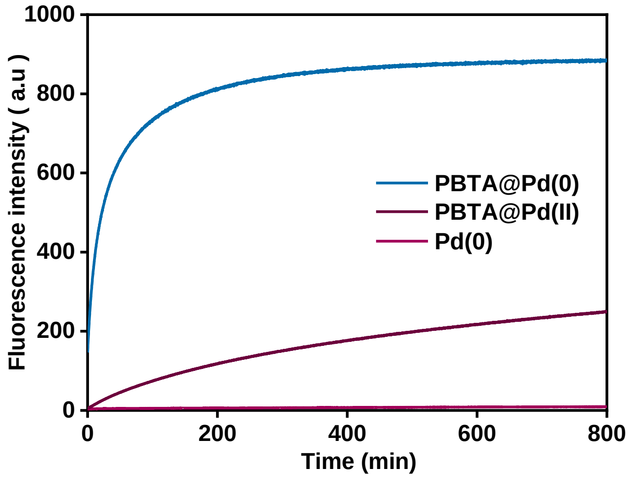

Figure S15: Fluorescence kinetic profiles of **pro-rho (1)** activation using **PBTA@Pd(0), PBTA@Pd(II)** and bare **Pd(0)** in DMEM where formation of **rho (2)** was monitored at λ_{ex} = 485 nm and λ_{em} = 520 nm, reaction conditions: [**1**] = 30 μM, [Pd(0) or Pd(II)] = 30 μM, [**P**] ~ 0.142 mg/mL, T = 37 °C, in DMEM.

Cell experiments

Assessment of cell viability: HepG2 cells were cultured and seeded in Dulbecco's modified Eagle's medium (DMEM) supplemented with 10% fetal bovine serum (FBS) and phenol red. Cytotoxicity of **P@Pd(0)** was studied using the cell counting kit-8 (CCK-8) assay. A 96-well plate was used seed HepG2 cells. Wells were filled with 100 μL of cell suspension containing 8000 cells. The plate was then placed in an oven at 37 °C with 5% CO_2 flow for 24 h. Then,

the **P@Pd(0)** to be tested was added to the cells by varying Pd(0) from 20 – 100 µM. The amount of stock solution volume to be added was first removed from the well to keep concentrations constant. Followingly, the plate was placed in the oven. After 24 h, the medium was removed and 100 µL DMEM with 10% CCK8 were added to each well. The plate was then placed back in the oven at 37 °C for 2 to 4 h. The absorbance of each well containing cells was measured at 450 nm at the microplate reader. Cell viability was determined as a fold change of the absorbance with respect to untreated cells. Error bars represent the standard deviation of 3 different wells incubated with same sample.

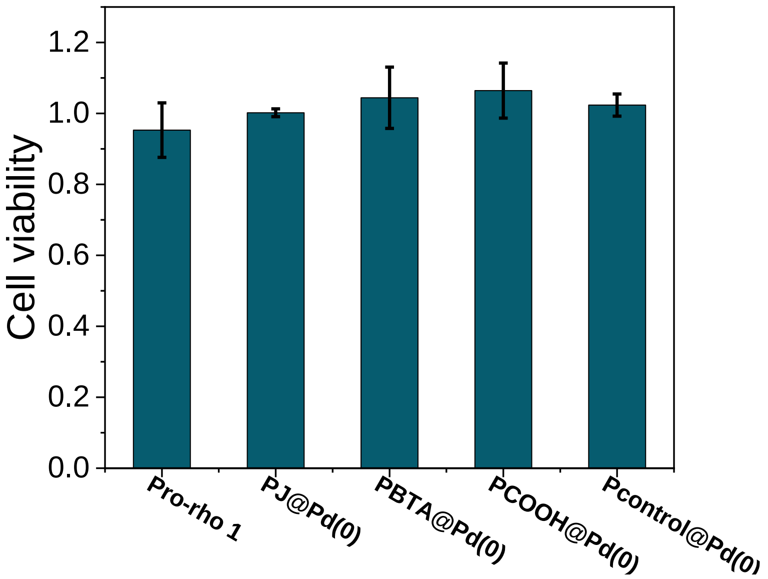

Figure S16: Cytotoxicity of Pd based SCPNs (Pd concentration: 60 µM) and pro-rho **1** (25 µM) incubated with HepG2 cells respectively for 48 h.

Procedure for pro-rhodamine activation in HepG2 cells: Cultured HepG2 cells were seeded in a µ-Slide 18 well from Ibidi. Wells were filled with 100 µL of cell suspension containing 8000 cells. Cells were incubated with **P@Pd(0)** and **1** for the incubation time as mentioned from the corresponding stock solutions. Later, the µ-Slide was placed back in the oven. The cells were then monitored in a confocal microscope at an excitation wavelength of 485 nm. For the control experiment, only pro-rho **1** was incubated. It is good to note here that the hydrophobic **pro-rho 1** by itself tends to precipitate in aqueous solution at concentrations > 100 µM as large aggregates, which reduces its ability to enter cells.

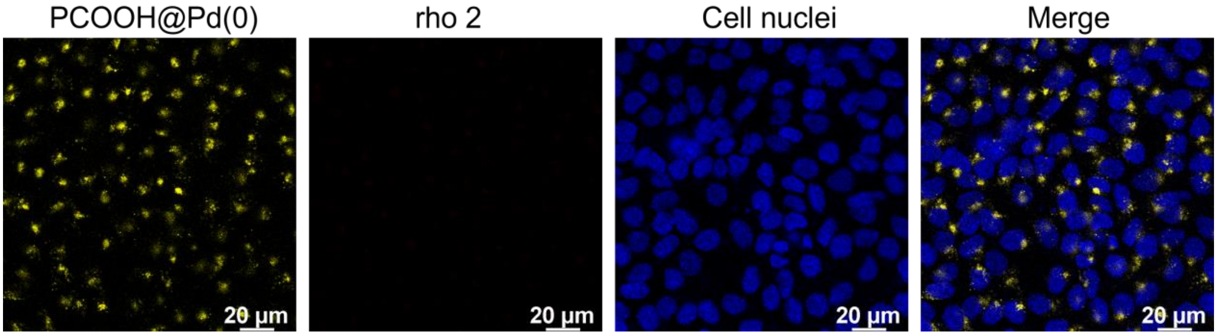

Figure S17: Confocal images of **pro-rho (1)** activation by **PCOOH@Pd(0)** inside HepG2 cells. The yellow and blue colors indicate **PCOOH@Pd(0)** and cell nuclei stained with Hoechst, respectively.

Procedure for pro-drug activation in HepG2 cells: The procedure was followed as explained above for pro-5FU **3**, pro-DiFU **5**, pro-dox **6** as substrates. After the indicated reaction times the compound containing medium was removed and 100 µL DMEM with 10% CCK-8 was added to each well. The plate was then placed back in the oven at 37 °C for 2 to 4 h. The absorbance of each well containing the cells was measured at 450 nm at the microplate reader. Cell viability was determined as explained before.

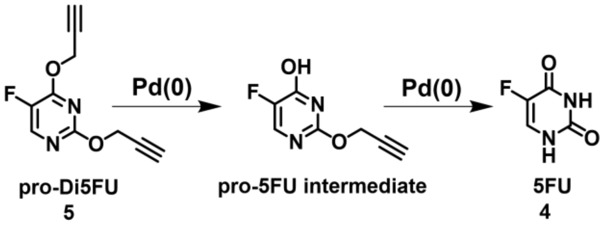

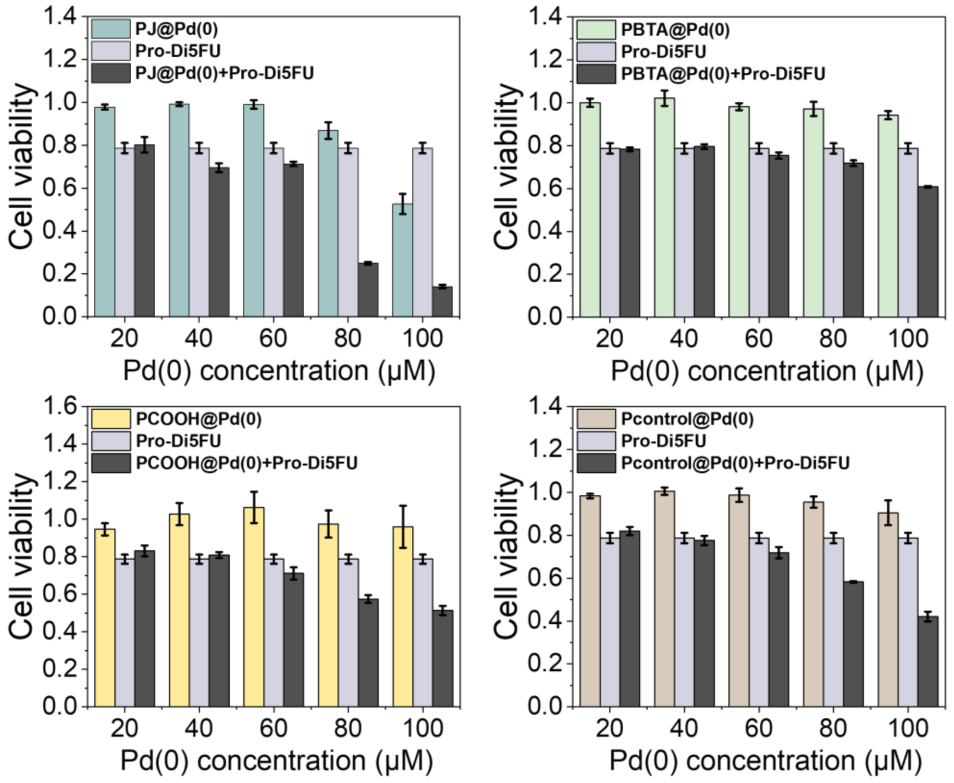

Figure S18: Activation of pro-Di5FU **5** (100 µM) by Pd(0) based SCPNs in the presence of HepG2 cells (incubation time: 48 h).

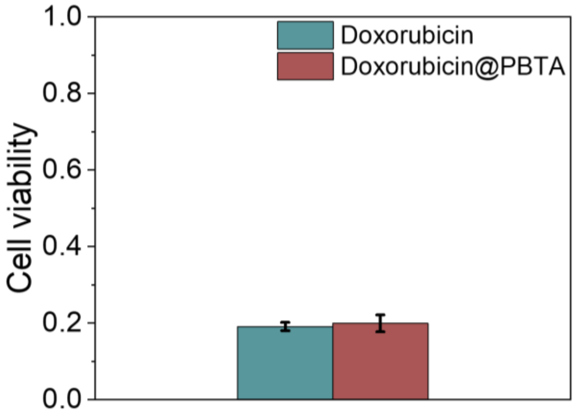

Figure S19: Cytotoxicity of doxorubicin (10 μM) and doxorubicin (10 μM) encapsulated by **PBTA** (0.5 mg/mL), incubated with HepG2 cells respectively for 48 h.

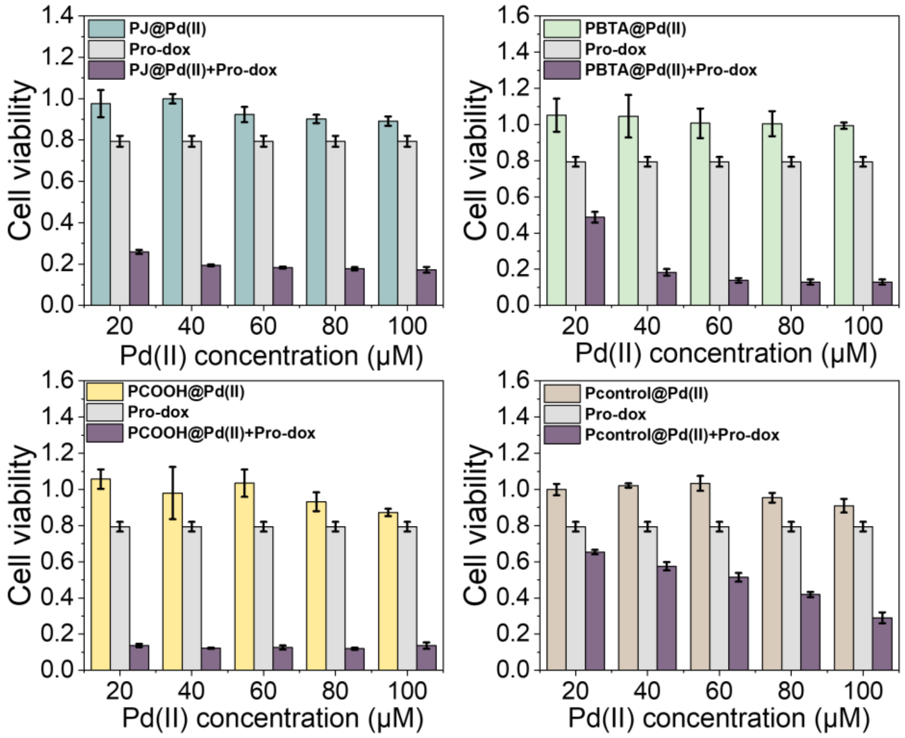

Figure S20: Activation of pro-dox **6** (100 μM) by Pd(II) based SCPNs in the presence of HepG2 cells (incubation time: 48 h).

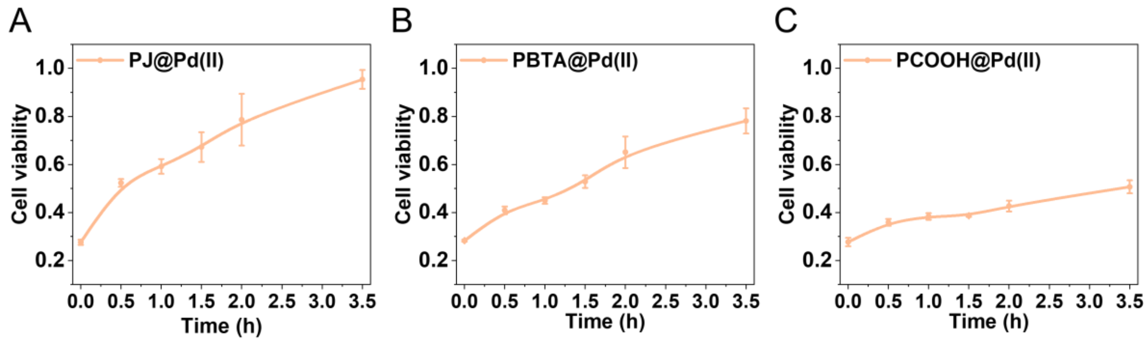

Figure S21: Incubation of **P@Pd(II)** ([Pd(II)]: 80 μM) with HepG2 cells for 0 h, 0.5 h, 1 h, 1.5 h, 2 h and 3.5 h prior to adding pro-dox **6** (10 μM) for the deactivation study.

3. References

(1) ter Huurne, G. M.; de Windt, L. N. J.; Liu, Y.; Meijer, E. W.; Voets, I. K.; Palmans, A. R. A. Improving the Folding of Supramolecular Copolymers by Controlling the Assembly Pathway Complexity. *Macromolecules* **2017**, *50* (21), 8562–8569.

(2) Sathyan, A.; Croke, S.; Pérez-López, A. M.; de Waal, B. F. M.; Unciti-Broceta, A.; Palmans, A. R. A. Developing Pd(II) Based Amphiphilic Polymeric Nanoparticles for pro-Drug Activation in Complex Media. *Mol. Syst. Des. Eng.* **2022**, *7* (12), 1736–1748.

(3) Deng, L.; Albertazzi, L.; Palmans, A. R. A. Elucidating the Stability of Single-Chain Polymeric Nanoparticles in Biological Media and Living Cells. *Biomacromolecules* **2022**, *23* (1), 326–338.

(4) Adam, C.; Bray, T. L.; Pérez-López, A. M.; Tan, E. H.; Rubio-Ruiz, B.; Baillache, D. J.; Houston, D. R.; Salji, M. J.; Leung, H. Y.; Unciti-Broceta, A. A 5-FU Precursor Designed to Evade Anabolic and Catabolic Drug Pathways and Activated by Pd Chemistry in Vitro and in Vivo. *J. Med. Chem.* **2022**, *65* (1), 552–561.

(5) Bray, T. L.; Salji, M.; Brombin, A.; Pérez-López, A. M.; Rubio-Ruiz, B.; Galbraith, L. C. A.; Patton, E. E.; Leung, H. Y.; Unciti-Broceta, A. Bright Insights into Palladium-Triggered Local Chemotherapy. *Chem. Sci.* **2018**, *9* (37), 7354–7361.

(6) Miller, M. A.; Askevold, B.; Mikula, H.; Kohler, R. H.; Pirovich, D.; Weissleder, R. Nano-Palladium Is a Cellular Catalyst for in Vivo Chemistry. *Nat. Commun*. **2017**, *8* (1), 1–13.
